# Establishing Sterility Assurance for *Bacillus canaveralius* 29669 Spores Under High Heat Exposure

**DOI:** 10.3389/fmicb.2022.909997

**Published:** 2022-07-11

**Authors:** Zachary Steven Dean, Michael DiNicola, Emily Klonicki, Scott Roberts, Brian Gregory Clement, Lisa Guan

**Affiliations:** ^1^Jet Propulsion Laboratory, California Institute of Technology, Biotechnology and Planetary Protection, Pasadena, CA, United States; ^2^Jet Propulsion Laboratory, California Institute of Technology, Systems Modeling, Analysis, and Architectures Group, Pasadena, CA, United States; ^3^Jet Propulsion Laboratory, California Institute of Technology, Materials Development and Manufacturing Technology Group, Pasadena, CA, United States

**Keywords:** Planetary Protection, heat microbial reduction, endospores, spacecraft bioburden, infrared heating, survival modeling, heat-resistant microorganisms

## Abstract

The ever-increasing complexity in critical spacecraft hardware and materials has led to the development of new microbial reduction procedures as well as to changes in established processes such as heat microbial reduction (HMR). In the space biology field of Planetary Protection, 500°C for 0.5 s is the current HMR recommendation to reduce microorganisms from flight hardware. However, more studies are needed to effectively determine the microbial reduction capability of high-temperature (more than 200°C), short-duration (under 30 s) heat exposures. One of the many recent microbial reduction bioengineering research avenues harnesses electromagnetic energy for microbial reduction, with previous investigations demonstrating that infrared heaters are capable of the short temperature ramp time required for rapid heating investigations above 200°C. Therefore, this study employed a 6 kW infrared heater to determine the survivability of heat resistant *Bacillus canaveralius* 29669 to high-temperature, short-duration infrared temperatures. While *B. canaveralius* 29669 spores can survive microbial heat reduction processes above 200°C, we found evidence suggesting that the 500°C for 0.5 s temperature sterilization specification for Planetary Protection should be updated. This research presents spore survival data and a corresponding model pointing to a re-evaluation of the recommended HMR exposure of 500°C for 0.5 s, while simultaneously meeting requirements on the forward biological contamination of solar system bodies and opening up design possibilities for future spacecraft hardware.

## 1. Introduction

Space exploration and interplanetary travel has revealed that terrestrial microbial life might be capable of survival in extraterrestrial environments (Chyba and Phillips, 2002; Saffary et al., 2002; McLean et al., 2006). As outer solar system missions continue to explore potentially habitable environments such as Mars and ocean worlds, planetary protection practices will be critical to prevent the deposition of terrestrial microorganisms, or forward contamination, and preserve data collected in future life-detection missions (Bruckner et al., 2009). The current era of Mars exploration has already motivated a number of recent advances in Planetary Protection as upcoming landed ocean worlds exploration requires heightened Planetary Protection sterilization protocols to ensure stringent cleanliness and reduce biocontamination (Pratt and Smith, 2020).

Bioburden, the microbial load on a product, consists of microorganisms that are capable of establishing a viable colony forming unit (International Organization for Standardization, 2010). These microorganisms can come from diverse sources such as human contact, air flow systems, material processing during spacecraft assembly, raw materials, and other environmental vectors that the material is exposed to (Whyte and Eaton, 2015). Microbial communities on spacecraft surfaces can range from fungi, bacterial spores, and dormant microorganisms, depending on the environmental conditions. These organisms vary widely in their ability to survive in extreme conditions. For example, researchers have studied the extremophile *Bacillus canaveralius* 29669, the current model organism for Planetary Protection heat sterilization studies, which was isolated from microbial fallout in clean rooms during the assembly of the Viking missions to Mars. Spores from this bacterial species are thirty times more resistant to dry heat than *Bacillus atrophaeus*, the universally-accepted heat microbial reduction indicator organism (Schubert and Beaudet, 2011). The exact reasoning for *B. canaveralius* 29669's heat resistance is unknown, but its genome contains a gene coding for a glycogen branching enzyme from hyperthermophile *Thermococcus kodakaraensis* which may contribute to its heat tolerance (Seuylemezian et al., 2018).

The Planetary Protection group at the NASA Jet Propulsion Laboratory is responsible for mitigating the bioburden of spacecraft destined for other moons or planets, such as Europa. Forms of Planetary Protection microbial reduction techniques used in missions include vaporized hydrogen peroxide, heat microbial reduction (HMR), and alcohol disinfection. These methods are implemented in cleanrooms and used on spacecraft in order to reduce the biological matter that may contaminate the hardware (Pillinger et al., 2006). In addition to bioburden reduction capabilities, Planetary Protection must also take into account hardware compatibility to microbial reduction techniques when determining the optimal disinfection techniques and standards.

HMR is a microbial reduction technique where spacecraft and mechanical components are kept at high temperatures under controlled humidity for a predetermined exposure time. HMR is the most commonly used approach for reducing bioburden on spacecraft hardware. NASA recently revised HMR specifications to provide D-values, which are the defined times and temperatures required to reduce viable bacteria by 90%, for temperatures up to 200°C (NASA Science Mission Directorate, 2008). *D*-values for higher temperatures are derived by Viking-era (1970s) findings, which are not appropriate for predicting lethality above a 3-log reduction (Shirey et al., 2017). The NASA HMR specifications for absolute sterility, defined as a 12-log reduction, requires hardware to undergo HMR at 500°C for 0.5 s (NASA Science Mission Directorate, 2008).

Previous investigations evaluating heat sterilization techniques have demonstrated that electromagnetic radiation is capable of high temperature sterilization (e.g., 500°C in 30 s), and that the heating, not the infrared wavelength photochemical effects, cause significant microbial killing (Kempf et al., 2005). Despite significant research on heat microbial reduction, there is still a lack of data for the high-temperature, short-duration heat exposures that will assist in determining the minimal temperature and exposure time necessary for the efficient sterilization. In this study, an infrared (IR) halogen lamp system was designed, built, and employed to test survival of *B. canaveralius* 29669 spores exposed to temperatures above 200°C for <30 s. *B. canaveralius* 29669 spores are used as the model microorganism for this study due to its high heat resistance and its role in informing current NASA requirements (NASA Science Mission Directorate, 2017). The objective is to use experimental results and mathematical modeling to bound the time-temperature at which the Sterility Assurance Level (SAL), or probability that one or more microorganisms survive, reaches 10^−6^ (Swenson, 2012). As part of this analysis, we will also investigate the probability that an individual microorganism survives, *s*, as well as *D*-values and *z*-values associated with these experiments.

## 2. Materials and Methods

### 2.1. Preliminary Spore Evaluation and Spore Strain Selection

The *B. canaveralius* 29669 organisms used in this study were obtained from the American Type Culture Collection (Manassas, VA) with spore production carried out following previously described methods (Schubert and Beaudet, 2011). Six sub-cultures derived from the original *B. canaveralius* 29669 parent culture were characterized in this study's investigation. The six sub-cultures were tested to identify the batch most closely matching the *D*-value from the *B. canaveralius* 29669 culture used to determine the 2008 NPR Planetary Protection heat sterilization parameters, 39.7 min at 150°C (Kempf et al., 2008; NASA Science Mission Directorate, 2017). Five microliters of 1.5 × 10^6^ *B. canaveralius* 29669 spores per μL suspended in deionized water were spotted onto the bottom of thin-walled, flat-bottom stainless steel thermal spore exposure vessels, or TSEVs, that are 1.27 cm internal diameter, 10.16 cm long, and a wall thickness of 0.025 cm (Supplementary Figure 1A; Kempf et al., 2008). After spotting with spores, the TSEVs were then dried in a biological safety hood overnight (8 h minimum). Following overnight drying, the TSEVs were then placed in a lyophilizer (Millrock Technology, Inc., Kingston, NY) for an hour. The spore heat survival experiments were then executed in a high-temperature thermometer calibration oil bath (Model 6330, Hart Scientific, American Fork, UT, Supplementary Figure 1B) filled with a silicone oil (Hart bath fluid #5017, Dow Corning silicone oil type 710, Hart Scientific, American Fork, UT). The bath temperature was preheated to 150°C for 30 min and held to within ±0.05° of the target temperature during the exposures with continuous oil stirring to facilitate equal temperature distribution to all TSEVs. Thermocouples were calibrated through immersion in the thermometer calibration bath prior to use. The inoculated TSEVs were placed under 1.5 Torr vacuum conditions using a silicone rubber septum (ST-495, Specialty Silicone Products, Inc., Ballston Spa, NY) and an aluminum crimp-seal (20 mm, Wheaton Science Products, Millville, NJ); this allowed for exposure of the spores in a controlled humidity environment. The septum was penetrated with a needle (22-gauge, side-hole, Hamilton Company, Reno, NV) and evacuated with a vacuum pump (TriScroll 300, Varian Vacuum Technologies, Lexington, MA). Temperature from the TSEV thermocouples was recorded using an Agilent 34970 data logger (Agilent Technologies, Inc., Santa Clara, CA) and a custom LabView program (version 6.0, National Instruments, Austin, TX). After completing the heat exposure, the TSEVs were removed and immediately placed in an ice bath until cooled to room temperature.

Following cooling and after removing the crimp seal and septum, 1 mL of sterile deionized water was added to each TSEV to acquire spores. The TSEVs were then resealed and vortexed for 10 s before sonicating at 25 kHz for 2 min. The TSEVs were then vortexed again for 10 s. The samples were then serially diluted from 10^−1^ to 10^−5^ with 100 uL of the original TSEV spore sample into 900 uL of sterile water and 1 mL of each subsequent dilution (into 9 mL of DI water) was plated in tryptic soy agar (Difco Tryptic Soy Agar, BD Biosciences, Franklin Lakes, NJ). The colonies were counted after four days of incubation at 37°C. Unheated samples were processed as positive controls with TSEVs lacking organisms serving as negative controls.

### 2.2. High Heat Infrared Lamp Experimental Design and Setup

**Figure 1:**
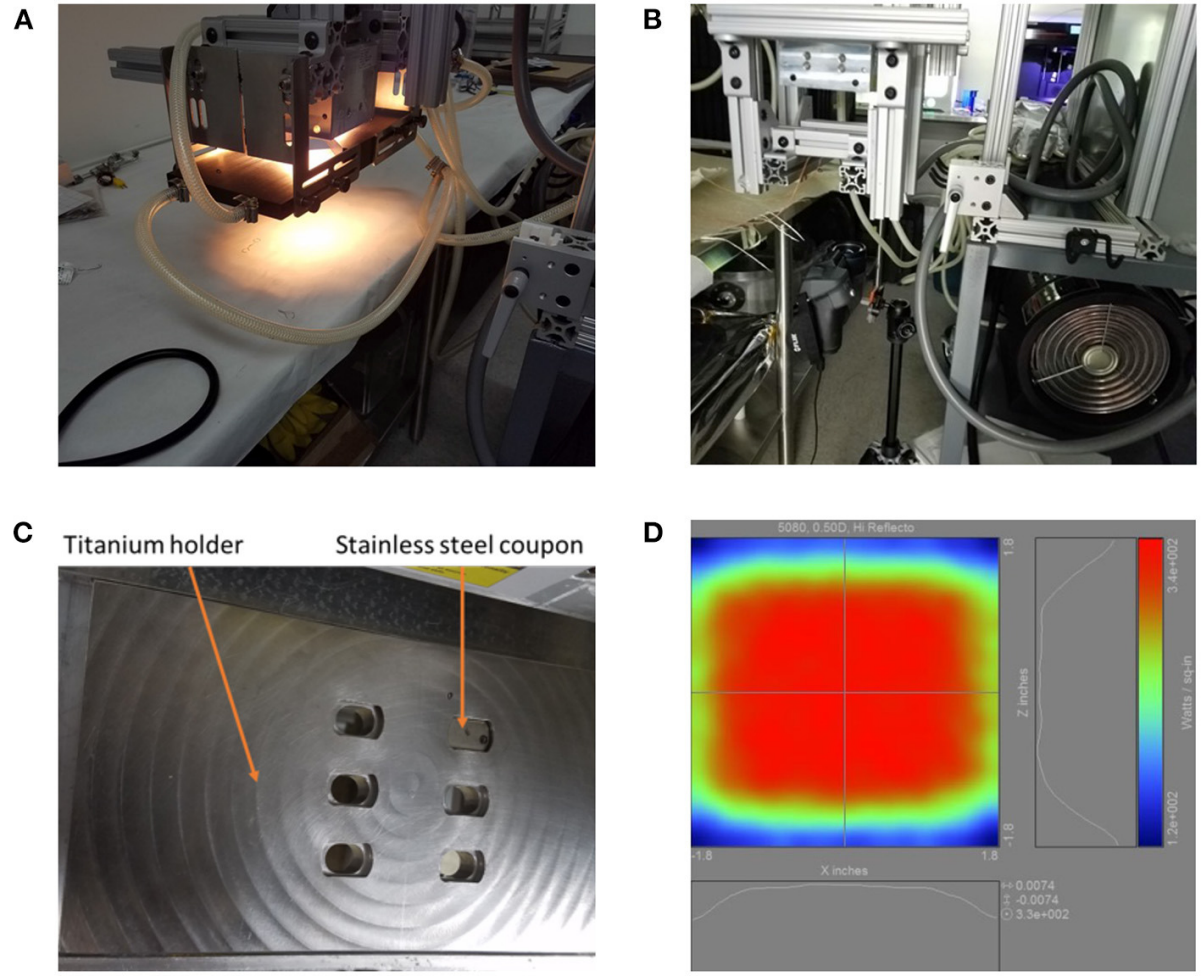
The 5080 high temp infrared heater lamp setup. **(A)** The lamp is shown turned on. White light appears in the photo, but the majority of the light originating out of the bulbs is infrared. **(B)** The IR heat exposure setup including the FLIR T650sc camera, 5080 high temp IR heater lamp, and the titanium holder support structure consisting of 80/20 aluminum. **(C)** The titanium coupon holder with one stainless steel coupon inserted. **(D)** Projected heating profile for objects under the lamp.

The 5080-06-02-6kW-24 High Temp IR Heater (Precision Control Systems, Inc., Eden Prairie, MN) used in this experiment includes six 1,000 W T3 short wavelength halogen lamps (Figure 1). The IR heater's ability to transfer infrared energy to the coupons was characterized prior to exposing coupons and spores to heat by using K-type thermocouples spot welded to stainless steel coupons in each position within the titanium coupon holder (Supplementary Figure 2). In addition, the ability of the IR heater to warm a single location on the holder for 5–8 s was also measured (Supplementary Figure 3).

#### 2.2.1. *Bacillus canaveralius* 29669 Spore Emissivity on Kapton Stainless Steel Coupons

**Figure 2:**
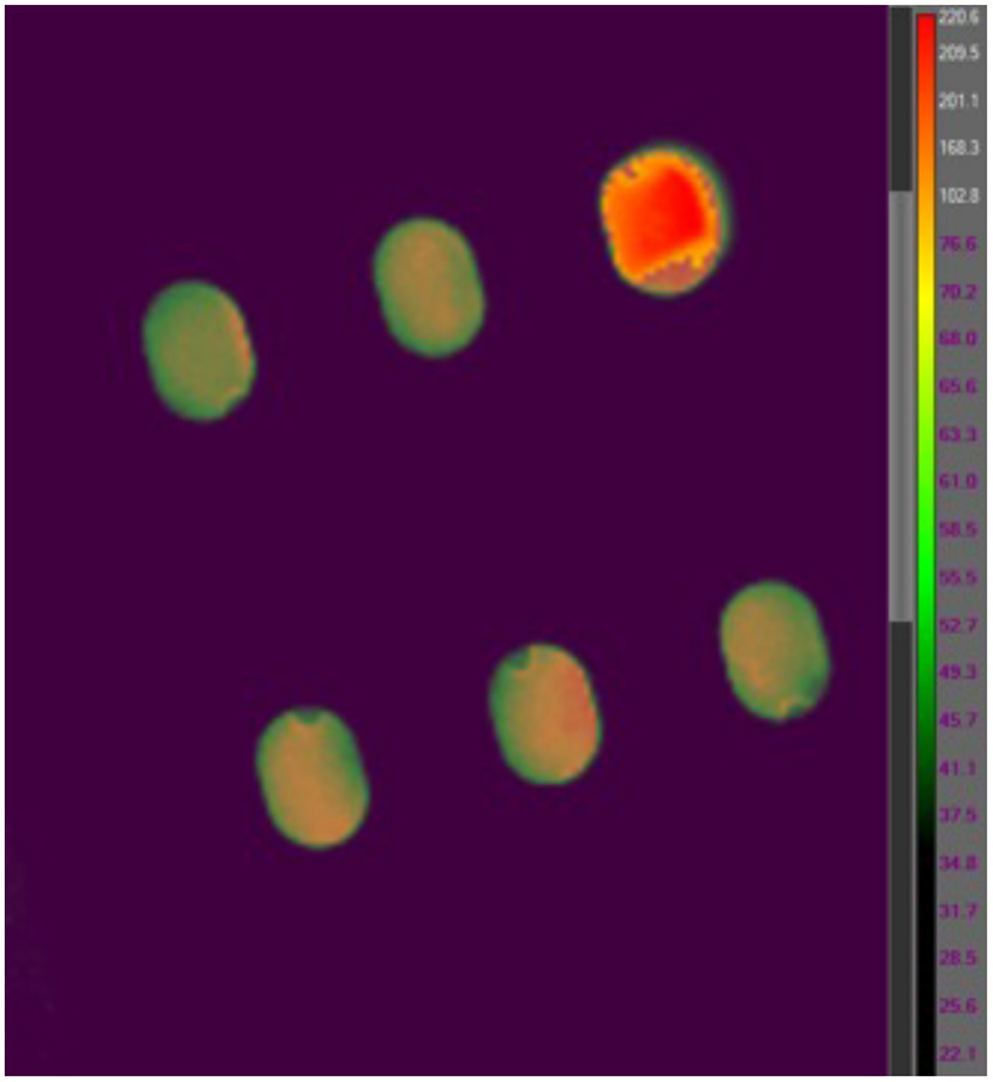
An example coupon IR heating image from the FLIR T650sc IR camera viewed in FLIR ResearchIR 14. The coupon measured is located in the upper-right corner of the image. The other five coupon slots are out of focus, and the temperatures are not representative.

While polyimide tape (Kapton) emissivity is 0.95 and stainless steel type 301 emissivity is 0.54–0.63 at a temperature of 300 K (Emissivity Coefficients Materials, 2003) the emissivity of *B. canaveralius* 29669 spores is not known. The emissivity of carbon at 300 K is 0.81, and because spores are made largely of carbon this is a reasonable rough estimate. However, to gather more accurate information on *B. canaveralius* 29669 spore emissivity, dried *B. canaveralius* 29669 spores at a known temperature were measured with the T650sc camera (Figure 2). An accurate emissivity leads to a more precise infrared record of the *B. canaveralius* 29669 spore temperature during heating. First, a 5 μL droplet of *B. canaveralius* 29669 spores was spotted on polyimide-taped stainless steel coupons. Then, the coupons were placed in a dry block heater that was heated to 150°C. After the coupon temperature reached steady state, the temperature of the spore spots was measured with the IR camera, and the emissivity was adjusted until the temperature shown on ResearchIR for the spore spots reached the estimated 150°C temperature (Figure 2). The experiment showed that the spore spot on Kapton-taped stainless steel coupons produces an emissivity of 0.914, which falls within expected values.

#### 2.2.2. Coupon Preparation and Inoculation for IR Heating

For each exposure, custom (8 mm wide by 13 mm long by 0.5 mm thick) stainless steel coupons (Supplementary Figure 4A) were flattened and high temperature polyimide tape (1/4” wide) was placed on the coupons. The taped coupons were autoclaved at 121°C for 15 min in a sealed glass petri dish prior to inoculating with spores. Inoculation of spores was performed in a biosafety hood by first diluting the spores to 8 × 10^5^ spores/μL using deionized water, then vortexing the spores for 5 s. Diluting spores is done to prevent spore overlap and allow for a monolayer to form during the drying and heating process. A 5 μL spore spot was then pipetted onto the center of the polyimide tape and the spores were dried overnight (at least 8 h) in a biosafety hood.

#### 2.2.3. High Heat Infrared Exposure and Imaging

Inoculated coupons were placed in the titanium holder (Figure 1) with the spores facing away from the 5080 IR lamp. A T650sc IR camera (FLIR Systems, Inc., Wilsonville, OR) was placed directly under the titanium holder to image the temperature change of the spores throughout the exposure at 30 Hz (one image per 0.033 s). The 5080 High Temp IR Heater was used as the IR source (Figure 1). The titanium coupon holder with coupons was placed 0.2 inches from the IR heater quartz window and heating elements during the spore exposure experiments. To irradiate the samples, the IR lamp was used at 6 kW. Once the desired heating time was reached, the lamp was turned off as well as physically displaced from the coupon holder to prevent further heating. For survivor ratio estimates, coupons were immediately placed in sterile 10 mL glass screw-cap test tubes; for fraction negative (binary, growth/no-growth test) measurements (Harris and Skopek, 1985), coupons were placed into a 10 mL glass screw-cap test tube with 10mL pre-sterilized (autoclaved at 121°C for 15 min) trypticase soy broth (Bacto Tryptic Soy Broth, BD Biosciences, Franklin Lakes, NJ). All IR images were analyzed using FLIR ResearchIR 14. To measure coupon spot temperatures the emissivity was set to 0.914, and 16 pixels were averaged across the center of each coupon with the coupon distance from the camera fixed during exposures to maintain coupon size and image resolution throughout the experiment.

#### 2.2.4. Post-exposure Spore Processing and Culture

After each IR heat exposure, coupons were processed in a biosafety hood. For survivor ratio analysis, 1 mL of sterile, deionized water was added to each tube, submerging each coupon. The tubes were vortexed for 10 s, then sonicated for 2 min and vortexed a second time for 10 s to remove the spores from the surface of the coupons. The samples were then diluted from 10-1 to 10-5 of the original concentration with 1 mL of the initial sample diluted into 9 mL of sterile water. One microliter of each dilution was then plated in tryptic soy agar (Difco Tryptic Soy Agar, BD Biosciences, Franklin Lakes, NJ). The colonies were counted after 24, 48, and 72 h of incubation at 37°C. Unexposed (unheated) samples were processed as positive controls, and coupons without spores were processed as negative controls.

For fraction negative (binary, growth/no-growth test) analysis, the same procedure was followed as with survivor ratio tests; however, coupons were transferred to 10 mL screw-cap test tubes containing tryptic soy broth immediately following exposure. Fraction negative turbidity compared to positive control (unexposed) samples and negative control (coupon blank) samples distinguished complete sterilization from surviving populations after incubation at 37°C for 72 h with shaking at 100 RPM.

### 2.3. Mathematical Method

The mathematical model developed for this study aims to estimate the probability of sterilization under various heat exposure conditions.^1^ It does so by defining a model, within a probabilistic framework, of the processes involved with generating the data observed from the HMR experiments. Rather than assuming fixed experimental parameters such as the starting population and protocol recovery efficiency, the model takes these parameters as distributions based on data collected from control experiments. Direct observation (CFU counts, growth/no growth) from the heat exposure experiments were then used to further calibrate the distributions of these parameters, particularly the *D*-value and *z*-value for *Bacillus canaveralis* 29669. The *D*-value is the time required at a specified temperature to reduce the number of viable organisms by 90%, and the *z*-value is the number of degrees required to decrease the *D*-value by 90%. The model performs this calibration using Bayes' Theorem, which essentially informs the model of the data generating process with the observed data to evaluate which *D*-values and *z*-values are more or less likely, relative to our knowledge prior to experimental observation. This information, together with a key assumption that the survival process is memoryless (to be discussed further in Section 2.3.4), allows the model to calculate the probability of individual microorganism survival and SALs for products with a given bioburden for a wide range of heat treatments of different time durations and temperatures.

The model has two main submodels to capture the experimental parameters: (1) a seeding model, developed in Section 2.3.1, that considers how many spores are on the coupon prior to heat treatment; and (2) a survival and recovery model that considers the exposure of the inoculated coupon to heat treatment and the survival and recovery of microorganisms from the coupon after heat treatment, first for survival ratio experiments in Section 2.3.2 and then for fraction-negative experiments in Section 2.3.3.

#### 2.3.1. Seeding Model

The experimental process described in Section 2.2.2 begins with the seeding of a coupon with a targeted number of ~10^6^ colony forming units (CFU). This involves taking a spore stock solution of volume *V* containing a *B. canaveralius* 29669 and applying sonication and vortexing to give the solution a uniform titer, τ. A volume *x* ≪ *V* of spore stock is then extracted *via* pipette from the stock solution and transferred onto a coupon. Given that the titer of the spore stock used for this study is on the order of 10^8^ CFU/mL and the volume extracted is 5 μL, the number of CFU in the solution, η = τ*V*, is very large compared to how many CFU are being extracted into the pipette, τ*x*. Losses due to CFU transfer to the coupon may result in a slightly lower but still uniform titer, τ′, of the solution transferred to the coupon than in the original solution.

All materials and reagents are tested for sterility according to manufacturer recommendations to avoid outside contamination that could introduce non-uniformities or heterogeneity of species into this process. Given the uniformity of the spore stock solution that is sampled, the probability, ρ, that an individual CFU is transferred from the solution to the coupon is the same for all CFU present in the solution, and $\rho =\frac{x\tau }{\eta }\times \frac{x\tau^{\prime }}{x\tau }=\frac{x\tau^{\prime }}{\eta }\ll 1$. Since sonication and vortexing obstruct microbial dependencies being formed between microorganisms, such as clumping or biofilm formation, it is reasonable to assume the transfer of individual CFUs from the spore stock to the coupon are independent; that is, the event that a given CFU is transferred from the stock solution to the coupon does not affect the chances of this event occurring for another CFU. Combining these independence and uniformity properties gives us that the number of CFU transferred from the spore stock to the coupon follows a binomial distribution with parameters η and ρ, where η is large and ρ is small. Note that the expected number of CFU transferred from the spore stock to the coupon is ηρ = *xτ*′, which is constant for a given inoculation level, and is approximately equal to the variance of CFU transferred from the spore stock to the coupon ηρ(1 − ρ), since 1 − ρ ≈ 1. It follows that the probability that *n* CFU are transferred from the original spore stock solution to a given coupon approximately follows a Poisson distribution with mean parameter λ ≈ *xτ*′:

(1) $$\begin{array}{r} p\left(n|\text{λ}\right)=e^{-\text{λ}}\frac{{\text{λ}}^{n}}{n!}. \end{array}$$

This study will treat λ as a parameter with a corresponding probability distribution. This distribution is initially estimated using control experiments and further calibrated simultaneously with other model parameters with observations from HMR experiments. Further discussion can be found in Supplementary Material (Section 1.2).

#### 2.3.2. Survival and Recovery Model: Survival Ratio Experiments

Let *S*(*T*), Φ and Θ_*d*_, *d* = 1, …, 5, be random variables taking values on the unit interval. *S*(*T*) will represent the probability that an individual microorganism survives heat treatment *T*, and is referred to as the survival function. This function is parameterized by the *D*-value at the reference temperature *T*_0_ = 150°C and *z*-value. The *D*-value and *z*-value are parameters that are treated as random variables that take values δ(*T*_0_) and *z*. Supplementary Material (Section 1.1) contains the derivation of the survival function. The term, *T*, is a time-temperature profile described as a continuous real-valued function of time, *t*. We note that the model described in this study does not capture uncertainty in the time-temperature profile stemming from the emissivity factor calibration in discussed in Section 2.2.1. The variable Φ will represent the probability that an individual microorganism that survived heat treatment is extracted from the coupon and survives extraction into a water solution. Finally, Θ_*d*_ will represent the probability that an individual microorganism is transferred from the solution of extracted survivors into the sample taken from dilution of magnitude *d*, is plated and produces a CFU in growth medium, respectively. The dilution magnitude is an integer *i* in the set {1, 2, 3, 4, 5}; for example, a 10^−3^ dilution has a dilution magnitude of 3. Suppose for the time-being that we know the values of each of these random variables, denoted by *s*(*T*), ϕ, and θ_*d*_.

The original experiment described in Section 2.2.4 mostly performed one dilution series (i.e., a single replicate) for each coupon *m* exposed to heat treatment. The CFU observation from the first denumerable dilution of this series was then reported in the data. For computational reasons, we develop the model to incorporate data from the first replicate and a single dilution only, as prior studies have shown that incorporating more than one dilution does not improve estimates of starting value (Christen and Parker, 2020).

Suppose we observe *r*_*m*_ CFU from the dilution obtained from the *m*th coupon exposed to heat treatment *T*_*m*_ after plating. In what follows, we will let the function *d* map the *m*th coupon to the magnitude of its corresponding dilution magnitude, *d*(*m*). For each individual microorganism on coupon *m*, there are two mutually exclusive outcomes relevant to this study:

1. The microorganism survives heat treatment, is extracted from the coupon and survives extraction, is transferred *via* the dilution of magnitude *d*(*m*), plated and grows a CFU. This outcome occurs with probability *s*(*T*_*m*_) × ϕ × θ_*d*(*m*)_.
2. The microorganism either does not survive heat treatment, is not extracted from the coupon or does not survive extraction, or is not transferred *via* the dilution of magnitude *d*(*m*), plated and grown into a CFU; that is, the negation of (1). This outcome occurs with probability 1 − *s*(*T*_*m*_) × ϕ × θ_*d*(*m*)_.

Under outcome (1), we will say that the microorganism has survived and has been recovered. The model assumes that a surviving microorganism that has been plated will be counted as a single CFU in the data with probability equal to one, and that there is no error in counting. Moreover, if a microorganism ceases to survive at an earlier stage it will not become viable again at a later stage. For example, if a microorganism does not survive heat treatment or extraction, then there is zero probability that it will become viable during dilution or when placed in growth medium for culturing.

As with the seeding process, the design of the HMR experiments takes several measures to ensure uniformity of the materials and processes involved and to avoid outside contamination. These measures help ensure that all CFU follow the same survival function and have the same probability of recovery after exposure to heat treatment. Moreover, sonication and vortexing during extraction obstruct phenomena such as microorganism clumping, allowing us to model CFUs as being recovered independently of one another. Therefore, for modeling purposes we treat all microorganisms as independent of one another and identically distributed in terms of survival (during heat treatment and extraction) and transfer to dilutions for plating.

Now, assume there are *n* microorganisms seeded onto the coupon prior to heat treatment *T*_*m*_. The probability that a specific *r*_*m*_ microorganisms survive and are recovered (and *n* − *r*_*m*_ microorganisms do not survive or are not recovered) is


$$\begin{array}{l} {\left[s\left(T_{m}\right)\phi \theta_{d\left(m\right)}\right]}^{r_{m}}\times {\left[1-s\left(T_{m}\right)\phi \theta_{d\left(m\right)}\right]}^{n-r_{m}}, \end{array}$$

where *r*_*m*_ ≤ *n*. A specific set of *r*_*m*_ microorganisms can be chosen from the *n* microorganisms initially on the coupon in $\left(\begin{array}{c} n\\ r_{m} \end{array}\right)$ mutually exclusive ways. Hence, the probability that some set of *r*_*m*_ microorganisms survive and are recovered given that there were *n* microorganisms initially on the coupon prior to heat treatment is equal to

(2) $$\begin{array}{r} \;p\left(r_{m}|n,s\left(T_{m}\right),\phi ,\theta_{d\left(m\right)},T_{m}\right)\\ =\left(\begin{array}{c} n\\ r_{m} \end{array}\right){\left[s\left(T_{m}\right)\phi \theta_{d\left(m\right)}\right]}^{r_{m}}\times {\left[1-s\left(T_{m}\right)\phi \theta_{d\left(m\right)}\right]}^{n-r_{m}}, \end{array}$$

which is recognized as a binomial distribution with parameters [*n, s*(*T*_*m*_)ϕθ_*d*(*m*)_].

In order to capture the uncertainty present in the number of microorganisms seeded onto the coupon prior to heat treatment, we sum over all possible values of *n* using the model developed in Section 2.3.1. Hence, the probability of observing *r*_*m*_ CFU in the *m*th survival ratio experiment is

(3) $$\begin{array}{r} p\left(r_{m}|\text{λ},\delta \left(T_{0}\right),z,\phi ,\theta_{d\left(m\right)},T_{m}\right)\\ =\overset{\infty }{\underset{n=r_{m}}{\sum }}p\left(n|\text{λ}\right)p\left(r_{m}|n,s\left(T_{m}\right),\phi ,\theta_{d\left(m\right)},T_{m}\right)\\ =e^{-\text{λ}s\left(T_{m}\right)\phi \theta_{d\left(m\right)}}\frac{{\left(\text{λ}s\left(T_{m}\right)\phi \theta_{d\left(m\right)}\right)}^{r_{m}}}{r_{m}!}, \end{array}$$

where the second row follows from algebra. This is recognized as a Poisson distribution with mean parameter λ × *s*(*T*_*m*_) × ϕ × θ_*d*(*m*)_. Note that *s*(*T*_*m*_) is the survival function evaluated over time temperature profile *T*_*m*_. This function is parameterized by δ(*T*_0_) and *z*; these parameters are suppressed in the notation of *s*(*T*_*m*_) for readability.

Since experimental outcomes can be assumed to be independent of one another, the probability of observing the data **r** = (*r*_1_, …, *r*_*M*_) from all survival ratio experiments performed is

(4) $$\begin{array}{r} p\left(\text{r}|\text{λ},\delta \left(T_{0}\right),z,\phi ,\theta ,\text{T}\right)\\ \;=\overset{M}{\underset{m=1}{\prod }}p\left(r_{m}|\text{λ},\delta \left(T_{0}\right),z,\phi ,\theta_{d\left(m\right)},T_{m}\right), \end{array}$$

where ***θ*** = (θ_1_, …, θ_5_) and **T** = (*T*_1_, …, *T*_*M*_) that associate with each observation *r*_*m*_ the probability θ_*d*(*m*)_ and time-temperature profile *T*_*m*_. Here, coupon *m* was exposed to time-temperature profile *T*_*m*_ that resulted in *r*_*m*_ CFU being observed from a dilution of magnitude *d*(*m*), for *m* = 1, …, *M*.

Studies performed prior to performing high temperature HMR experiments allowed the development of distributions characterizing the parameters of the model, referred to as prior distributions. Independence was assumed among all parameters prior to making observations from the high heat experiments. While this assumption is not realistic, there exists enough data to uncover any correlations during fitting of the model and thereby avoid unnecessary computational time and further assumptions involved with including covariance matrices and positing associated prior distributions. Hence, the joint distribution prior to making observations from high heat experiments is $p\left[\text{λ},\delta \left(T_{0}\right),z,\phi ,\theta \right]=p\left(\text{λ}\right)\times p\left[\delta \left(T_{0}\right)\right]\times p\left(z\right)\times p\left(\phi \right)\times \prod_{i=1}^{5}p\left(\theta_{i}\right)$. The prior distributions for each parameter are further discussed in Supplementary Material (Section 1.2). Once prior distributions are defined and observations are made from survival ratio experiments, we employ Bayes' Theorem to calculate the posterior distribution of the parameters (λ, δ(*T*_0_), *z*, ϕ, ***θ***) using Equation (4):

(5) $$\begin{array}{rc} p_{SR}\left(\text{λ},\delta \left(T_{0}\right),z,\phi ,\theta |\text{r},\text{T}\right)\propto & p\left(\text{r}|\text{λ},\delta \left(T_{0}\right),z,\phi ,\theta ,\text{T}\right)\\ \;\times p\left(\text{λ},\delta \left(T_{0}\right),z,\phi ,\theta \right). \end{array}$$

Integrating *p*_*SR*_ from Equation (5) over λ, ϕ and ***θ*** gives the joint posterior distribution of δ(*T*_0_) and *z* from the survival ratio experiments:

(6) $$\begin{array}{r} \begin{array}{c} =\int_{\theta \in {\left[0,1\right]}^{5}}\int_{\phi =0}^{1}\int_{\text{λ}=0}^{\infty }p_{SR}\left(\text{λ},\delta \left(T_{0}\right),z,\phi ,\theta |\text{r},\text{T}\right)\text{dλ d}\phi \text{ d}\theta . \end{array} \end{array}$$

Finally, we can use Equations (3) and (5) to predict the number of microorganisms recovered from a similar survival ratio experiment (either an experiment already performed, or a new experiment) if provided a time-temperature profile, $\tilde{T}$, dilution magnitude, $\tilde{d}$, and similar coupon inoculation level. The probability that *r* ∈ ℕ microorganisms on a coupon survive after being exposed to heat treatment $\tilde{T}$ and are recovered through a dilution of magnitude $\tilde{d}$ is

(7) $$\begin{array}{r} p_{SR}\left(r|\tilde{T},\tilde{d},\text{r},\text{T}\right)\\ =\int p\left(r|\text{λ},\delta \left(T_{0}\right),z,\phi ,\theta_{\tilde{d}},\tilde{T}\right)\\ \times p_{SR}\left(\text{λ},\delta \left(T_{0}\right),z,\phi ,\theta |\text{r},\text{T}\right)\text{ d}\theta_{\tilde{d}}\text{ d}\phi \text{ d}z\text{ d}\delta \left(T_{0}\right)\text{ dλ}, \end{array}$$

where the limits of integration are suppressed in the expression above, but range from 0 to ∞ for λ, δ(*T*_0_) and *z*, and range from 0 to 1 for ϕ and $\theta_{\tilde{d}}$. We will use Equation (7) primarily for validation of the model.

#### 2.3.3. Survival and Recovery Model: Fraction-Negative Experiments

A simplification to Equation (4) can be made in order to consider fraction-negative experiments. First, suppose there are *M*′ fraction-negative experiments performed. After being exposed to time-temperature profile $T_{m}^{\prime }$, the observed outcome, $r_{m}^{\prime }$ is either 0 (no growth) or 1 (growth). Here, no growth occurs with probability ${\left[1-s\left(T_{m}^{\prime }\right)\right]}^{n}$, which is the probability that no microorganism survives; and the probability that growth occurs—that one or more microorganisms survive—is equal to one minus the probability of no growth. No extraction or dilution is performed, as CFU are cultured by directly transferring the heat treated coupon into a test tube of tryptic soy broth. Given that the mean number of CFU seeded onto the coupon is equal to λ, the probability of observing growth or no growth from fraction-negative experiment *m* is

(8) $$\begin{array}{r} p_{FN}\left(r_{m}^{\prime }|\text{λ},\delta \left(T_{0}\right),z,T_{m}^{\prime }\right)\\ =\overset{\infty }{\underset{n=r_{m}^{\prime }}{\sum }}p\left(n|\text{λ}\right){\left[1-{\left(1-s\left(T_{m}^{\prime }\right)\right)}^{n}\right]}^{r_{m}^{\prime }}{\left[1-s\left(T_{m}^{\prime }\right)\right]}^{n\left(1-r_{m}^{\prime }\right)}\\ =\{\begin{array}{cc} e^{-\text{λ}s\left(T_{m}^{\prime }\right)} & \text{if }r_{m}^{\prime }=0\\ 1-e^{-\text{λ}s\left(T_{m}^{\prime }\right)} & \text{if }r_{m}^{\prime }=1 \end{array}, \end{array}$$

which is recognized as a Bernoulli random variable with parameter $1-e^{-\text{λ}s\left(T_{m}^{\prime }\right)}$.

While it is reasonable, given the experimental design, to treat the outcomes of fraction-negative experiments as independent of one another, they are not, as a group, independent of survival ratio experiments. Survival ratio experiments were performed first to understand the mortality of *B. canaveralius* 29669 when exposed to heat treatment and hypothesize a narrower interval of temperatures that would sterilize the coupons. This hypothesis was then tested and validated by performing a set of fraction-negative experiments. We model this dependency, first, by incorporating knowledge gained from survival ratio experiments for the *D*-value and *z*-value survival parameters by using the joint *posterior* distribution of δ(*T*_0_) and *z* from the survival ratio experiments given by Equation (6) to inform a joint *prior* distribution on δ(*T*_0_) and *z* when bringing the fraction-negative experiments into the analysis. Secondly, we recognize that the design of experiments bias fraction-negative experiments to test at temperatures more likely to induce sterilization. Since λ, the only other parameter in the fraction-negative model, is uncorrelated with *D*-value and *z*-value parameters, further informing it with the fraction-negative observations of this study could artificially reduce its value, as we will be more likely to observe no growth the fewer spores there are on the coupon. To avoid this behavior, we let the distribution of λ be given by the posterior distribution *p*_*SR*_(λ ∣ **r**, **T**) from survival ratio experiments (calculated by integrating Equation (5) over all parameters except λ) and do not allow it to be further informed by the fraction-negative experiments.

Hence, using Equation (8), the probability of observing the data ${\text{r}}^{\prime }=\left(r_{1}^{\prime },\ldots ,r_{M^{\prime }}^{\prime }\right)$ from all fraction-negative experiments performed is

(9) $$\begin{array}{r} p_{FN}\left({\text{r}}^{\prime }|\delta \left(T_{0}\right),z,\text{r},\text{T},{\text{T}}^{\prime }\right)=\overset{M^{\prime }}{\underset{m=1}{\prod }}\int_{\text{λ}=0}^{\infty }p_{SR}\left(\text{λ}|\text{r},\text{T}\right)\\ \;\times p_{FN}\left(r_{m}^{\prime }|\text{λ},\delta \left(T_{0}\right),z,T_{m}^{\prime }\right)\text{dλ}, \end{array}$$

where $r_{m}^{\prime }\in \left\{0,1\right\}$ and ${\text{T}}^{\prime }=\left(T_{1}^{\prime },\ldots ,T_{M^{\prime }}^{\prime }\right)$ are the associated time-temperature profiles for each observation. Note the integration over possible values of λ is within the product to capture the uncertainty in the mean number of spores seeded onto each individual coupon.

Finally, using Equations (6) and (9) and applying Bayes' Theorem again, the joint posterior distribution of δ(*T*_0_) and *z* after performing the fraction-negative experiments is

(10) $$\begin{array}{rc} p_{FN}\left(\delta \left(T_{0}\right),z|\text{r},{\text{r}}^{\prime },\text{T},{\text{T}}^{\prime }\right)\propto & p_{FN}\left({\text{r}}^{\prime }|\delta \left(T_{0}\right),z,\text{r},\text{T},{\text{T}}^{\prime }\right)\\ \;\times p_{SR}\left(\delta \left(T_{0}\right),z|\text{r},\text{T}\right). \end{array}$$

#### 2.3.4. Predicting the Number of Microorganisms That Survive Heat Treatment and Comparison With the Sterility Assurance Level (SAL)

With the joint posterior distribution of δ(*T*_0_) and *z* in-hand, we can calculate the posterior predictive distribution of the number of microorganisms that survive a new heat treatment $\tilde{T}$ for a given bioburden. That is, if there are *n* microorganisms on a new coupon prior to heat treatment, then the probability that ${\tilde{n}}_{s}$ survive the heat treatment following time-temperature profile $\tilde{T}$ is given by

(11) $$\begin{array}{r} p\left({\tilde{n}}_{s}|n,\tilde{T},\text{r},{\text{r}}^{\prime },\text{T},{\text{T}}^{\prime }\right)\\ =\int_{\delta \left(T_{0}\right)=0}^{\infty }\int_{z=0}^{\infty }\left(\begin{array}{c} n\\ {\tilde{n}}_{s} \end{array}\right)s{\left(\tilde{T}\right)}^{{\tilde{n}}_{s}}{\left[1-s\left(\tilde{T}\right)\right]}^{n-{\tilde{n}}_{s}}\\ \times p_{FN}\left(\delta \left(T_{0}\right),z|\text{r},{\text{r}}^{\prime },\text{T},{\text{T}}^{\prime }\right)\text{ d}z\text{ d}\delta \left(T_{0}\right). \end{array}$$

This formulation will be used to evaluate the SAL for time-temperature profiles peaking above 270°C, since both survival ratio and fraction-negative experiments were performed at these temperatures. Since all fraction negative experiments saw peak temperatures greater than 270°C, we only use the survival ratio experiments for time-temperature profiles with peak temperatures between 200 and 270°C. More formally, when the peak temperature of $\tilde{T}$ is between 200 and 270°C and there are *n* microorganisms on a new coupon prior to heat treatment, then the probability that ${\tilde{n}}_{s}$ survive the heat treatment following time-temperature profile $\tilde{T}$ is given by

(12) $$\begin{array}{r} p\left({\tilde{n}}_{s}|n,\tilde{T},\text{r},\text{T}\right)\\ =\int_{\delta \left(T_{0}\right)=0}^{\infty }\int_{z=0}^{\infty }\left(\begin{array}{c} n\\ {\tilde{n}}_{s} \end{array}\right)s{\left(\tilde{T}\right)}^{{\tilde{n}}_{s}}{\left[1-s\left(\tilde{T}\right)\right]}^{n-{\tilde{n}}_{s}}\\ \times p_{SR}\left(\delta \left(T_{0}\right),z|\text{r},\text{T}\right)\text{d}z\text{d}\delta \left(T_{0}\right). \end{array}$$

Note that there is an element of extrapolation when applying this formulation to diverse time-temperature profiles not used in the experiments, as well as to other metallic surfaces different than the steel coupons used in this study. The extrapolation to differently shaped time-temperature profiles (that are within a similar range of temperatures as those used in this study) is reasonable if the survival probability of an individual microorganism is *memoryless*. What this means is that the model assumes that survival of a microorganism over any time interval on the time-temperature profile depends on the temperature experienced on, and time duration of that time interval, but not on the heat treatment the microorganism has already survived up to that time. This characteristic is implicitly assumed in practice when modeling survival with conventional *D*-values. In this study, we make this explicit in the derivation of the survival function (Supplementary Material, Section 1.1) and further claim that the assumption of memorylessness is reasonable because the temperatures to which *B. canaveralius* 29669 spores are exposed to in this study render biological repair mechanisms ineffective.

We can now respond to the objective of this analysis in the following way:

Let $\tilde{T}$ be a constant time-temperature profile taking the temperature T for some amount of time, τ, that is <30 s. Since $\tilde{T}$ is fully specified by the pair (T, τ), we let $\tilde{T}=\left(\text{T},\tau \right)$ and we find all (T, τ) such that

(13) $$\begin{array}{r} 1-p\left({\tilde{n}}_{s}=0|n,\left(\text{T},\tau \right),\text{r},{\text{r}}^{\prime },\text{T},{\text{T}}^{\prime }\right)=10^{-6},\text{if T}\geq \text{270} \end{array}$$

(14) $$\begin{array}{r} 1-p\left({\tilde{n}}_{s}=0|n,\left(\text{T},\tau \right),\text{r},\text{T}\right)=10^{-6},\text{if T}<\text{270}. \end{array}$$

To respond to the objective of this study, Equations (13) and (14) will be computed and tabulated for a range of bioburdens, *n*.

## 3. Results

### 3.1. Characterizing the *Bacillus canaveralius* 29669 Spore Stock

Seven *B. canaveralius* 29669 spore strains were tested for their ability to survive heat prior to evaluating the microbial reduction effects of IR heat on *B. canaveralius* 29669 spores. The spores were judged based on their heat resistance at 150°C compared to the *B. canaveralius* 29669 spores that the NASA Procedural Requirements are based on Schubert and Beaudet (2011) and NASA Science Mission Directorate (2017). Of the seven isolates tested, M4-6 was the closest match to the survival curve (direct enumeration) from the NASA Procedural Requirements-based strain based on its survival at 150°C (Schubert and Beaudet, 2011). Supplementary Figure 5 shows the survival curve of M4-6 spores at 150°. The *D*-value of the M4-6 isolate was 46.9 min in controlled humidity conditions, and the target value from the NASA Procedural Requirements strain was 39.7 min, suggesting an 18% increase resistance to dry heat at 150°C.

### 3.2. Characterization and Design of the High Heat Infrared Lamp Setup

The 5080 High Temp IR Heater's ability to transfer energy to the coupons was characterized prior to spore exposure. K-type thermocouples were spot welded to the stainless steel coupons and the coupons were placed in the custom-machined titanium holder with the thermocouple mounted on the coupon side facing away from the heater. The IR heater's ability to transfer heat to coupons based on the location within the coupon holder was first characterized as shown in Supplementary Figure 2. Peak heating was averaged over a 16-pixel area on the coupons for the data shown, and it is reported as resolved by the camera at 30 Hz. The same heating experiment produced 300°C temperatures on the top half (locations 1–3) of the coupon holder and 255°C temperatures on the lower half (locations 4–6) of the holder during 5 s of heating. This finding led to all further experiments, including all microbial reduction experiments, to be run on the top-left corner (position 1) of the titanium plate for increased precision in results as well as the highest heating rate.

The next two characterization runs used the top-left corner (position 1 in Supplementary Figure 2) slot of the titanium coupon holder to measure the ability of the IR heater to warm over 5, 6, 7, and 8 s as well as to evaluate the active heating and passive cooling capabilities of the system (Supplementary Figure 3). The 5, 6, 7, and 8 s heating times listed are the times from the point where the lamp is turned on until the lamp is turned off; however, the graph shows the coupon cooling rate as well. Five seconds of heating time reached an average of 172°C, 6 s reached an average of 218°C, 7 s reached an average of 263°C, and 8 s reached an average of 295°C; room temperature was held at 21°C for the experiment. Passive cooling from the highest temperature (295°C) to 121.1°C (the reference temperature where food sterilization is measured) took 22 s on average, so the entire heating process from initiating the system to finish was measured to take 30 s total.

### 3.3. Spore Monolayer on Coupons

**Figure 3:**
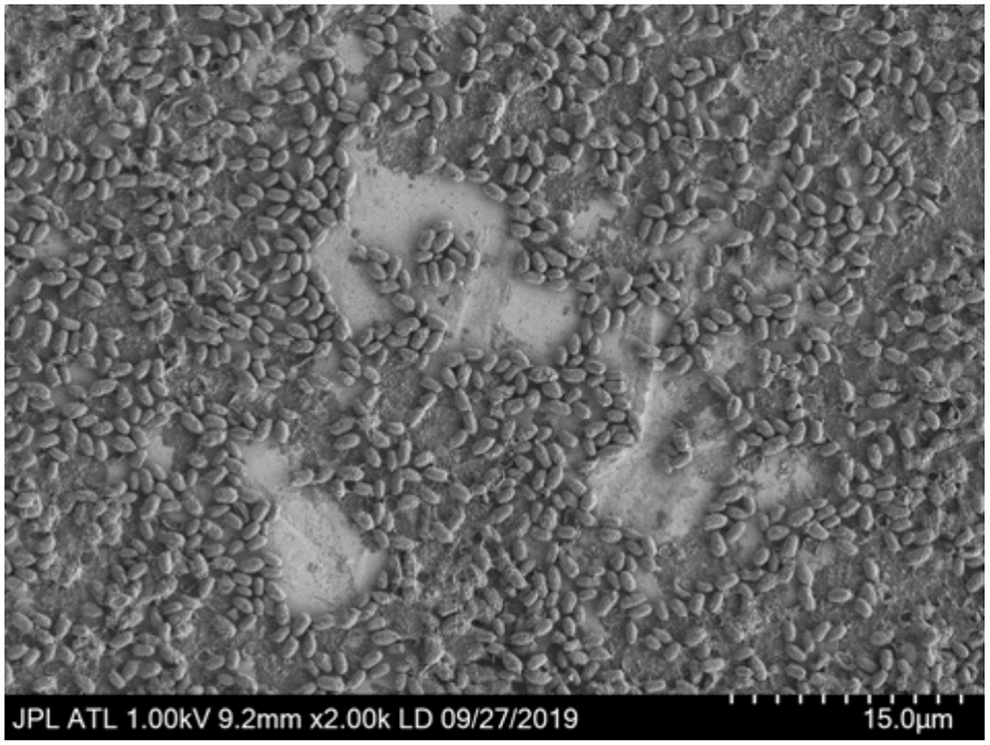
An electron microscope image of *Bacillus canaveralius* 29669 spores used to measure the spore size. 2 × 10^6^ spores per coupon were added before drying and lyophilizing. Based on the overlap in the image as well as a measured 10% coffee ring experience from 4 × 10^6^ spores/coupon dried on Kapton (Supplementary Figure 6), a minimal coffee ring effect is expected for the IR exposure experiments. 15 μm is the distance from the farthest left and farthest right hash on the bottom-right side of the image.

Overlapping spores is a concern for microbial reduction studies using heat conducted through a surface given they may insulate other spores from the heat exposure (Deng et al., 2005). To address this concern prior to the heat experiments, the spores were diluted to produce a monolayer of cells. In order to create the monolayer, the scanning electron microscope image (Figure 3) was used to measure the size of the *B. canaveralius* 29669 spores. The *B. canaveralius* 29669 spores were measured to be 1.494 μm long by 0.716 μm wide on average.

After determining the spore size and to make sure the spores fit into each spot placed on the stainless steel coupons, the contact angle of the spore-DI water storage solution on the coupon polyimide tape (Kizil et al., 2014) as well as the 5 μL volume of each spot were taken into account to determine the precise area needed to fit the spores: 8.24 mm^2^. From the dimensions needed, a maximum of 7.7 × 10^6^ spores were determined to fit within each 5 μL spot while still preventing an overlap. While this calculation does not take into account a coffee ring effect occurring during evaporation of the spore solution, the dried spore spot for the SEM image in Figure 3 shows 2 × 10^6^ spores/coupon, with minimal overlap occurring. An average of 4 × 10^6^ spores were placed onto the coupons for the IR heating experiments, and a maximum of 10% of the coffee ring effect was measured (Supplementary Figure 6); therefore, the coffee ring overlap effect on the spores used in the IR heating experiment was assumed negligible.

### 3.4. Infrared Spore Heat Exposure Experiments

*B. canaveralius* 29669 spores were exposed to short durations of infrared light in an ambient temperature of 21°C and an ambient humidity environment. The coupon temperature was measured with a FLIR T650sc infrared camera pointed directly at the coupons, and the coupon heat was evaluated with FLIR ResearchIR 14 software with the emissivity of the spores on the Kapton taped stainless steel coupons set to 0.914. Only heating above 150°C are used by the model developed in Section 2.3, as this is the temperature at which the *D*-value was studied for spore selection (Section 2.1). This temperature also provided an initial lower bound appropriate for a constant *z*-value survival model. Model results are not sensitive to reasonable changes in this value.

Testing stringent SALs directly (10^-6^, for example) is not empirically possible due to the logistics needed to effectively evaluate 10^12^ microorganisms on a single coupon at once (as is needed for a direct 10^-6^ SAL test; von Woedtke and Kramer, 2008). Instead, we use a lower density of microorganisms in testing combined with the mathematical model described in Section 2.3 to evaluate high heat exposures that satisfy various sterility assurance levels for a given bioburden.

Due to the low to zero survival numbers expected from the highest temperature experiments (268–334.2°C), fraction negative microbial reduction experiments, were also performed. The method used for the fraction negative experiments, which involved dropping the exposed coupon directly into nutrient broth, provided the sensitivity needed to detect even one surviving organism. Results from the fraction negative experiments can be found in Supplementary Table 1. No *B. canaveralius* 29669 extremophiles survived exposures to temperatures ranging from 268 to 334.2°C.

Finally, while evaluating *B. canaveralius* 29669 spore heat survival, the effect of the IR heating system ramp rate as a potential driver for cell death was also investigated. The survival of the *B. canaveralius* 29669 spores was not found to correlate with a temperature ramp rates in a linear model (Supplementary Figure 7, *R*^2^ = 0.081, *p*-value = 0.06).

### 3.5. Mathematical Model

**Figure 4:**
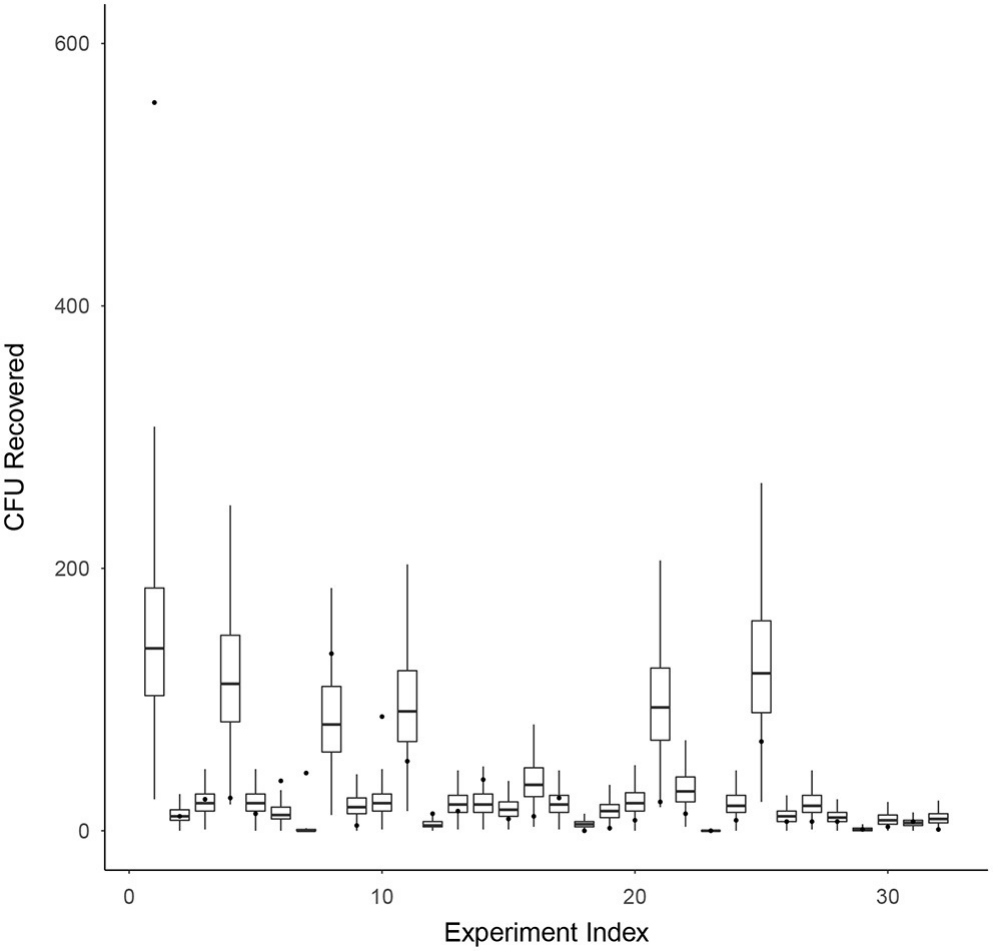
Model validation for survival ratio experiments using Equation (7). This figure compares the model's prediction of the number of CFU recovered (box-whisker plots) against the actual observed number of CFU recovered (black dots) on the vertical axis, for each experiment indexed by the integers 1–32 (horizontal axis). Box and whiskers show 50 and 95% credibility intervals, respectively, for the model's prediction of the number of CFU recovered from each experiment. The mean model prediction is shown with a horizontal line on each box-whisker plot.

Model validation for survival ratio experiments was conducted using Equation (7). Figure 4 compares the model's prediction of the number of CFU recovered (box-whisker plots) against the actual observed number of CFU recovered (black dots) on the vertical axis, for each experiment indexed by the integers 1–32 (horizontal axis). Box and whiskers show 50 and 95% credibility intervals, respectively, for the model's prediction of the number of CFU recovered from each experiment. The mean model prediction is shown with a horizontal line on each box-whisker plot. The model appears to be capturing the dispersion in the data well, with only 4 of the 32 experiments (experiment indexes 1, 6, 7, and 10) having actual observations falling outside the 95% credibility intervals of the model.

**Figure 5:**
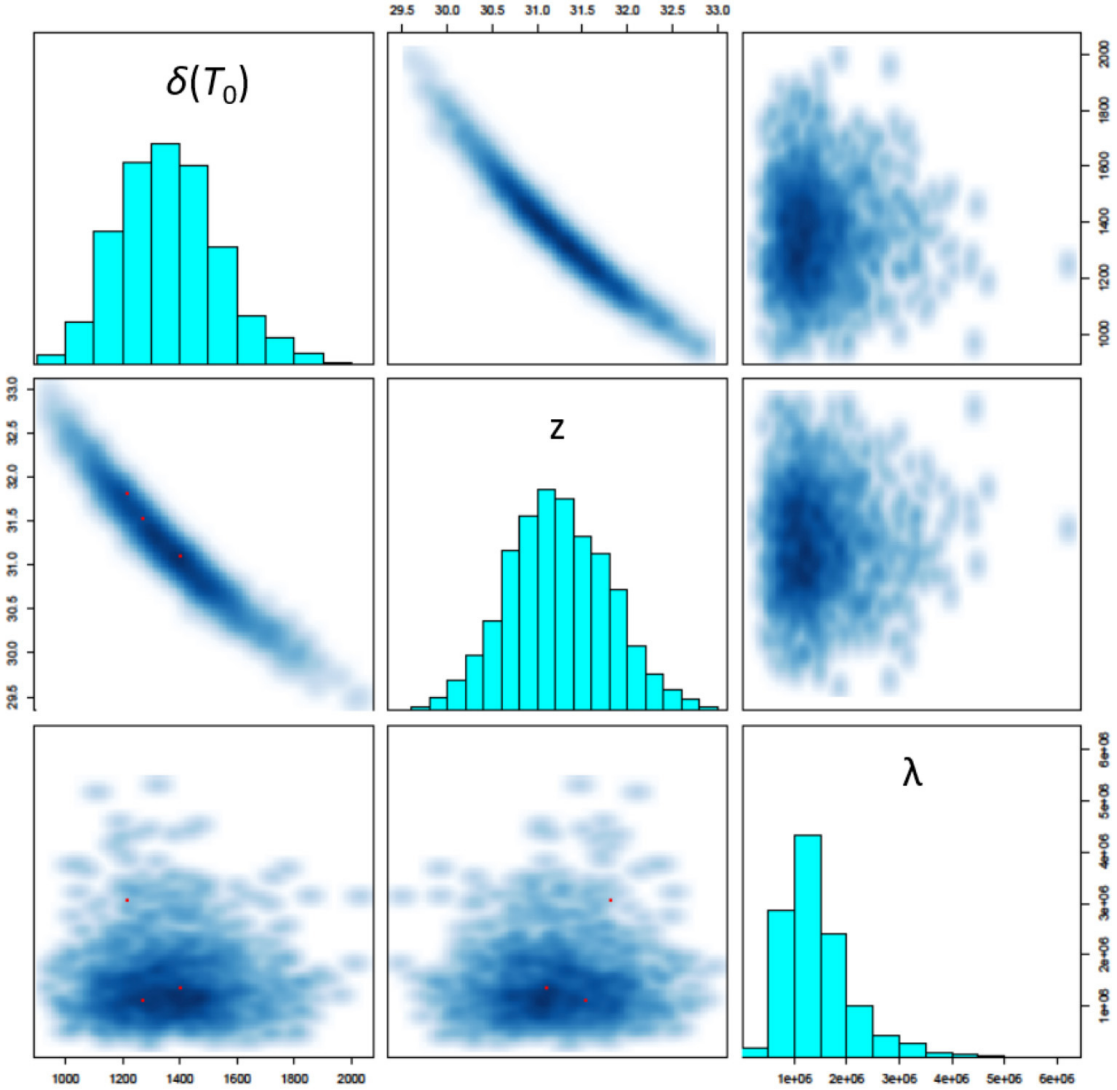
Pairs plot showing simulated posterior marginal distributions of model parameters along the diagonal and pairwise joint distributions off-diagonal. The three most critical parameters to assessing survival in the model are shown here: the *D*-value at 150°C [δ(*T*_0_)], the *z*-value (*z*) and the mean number of microorganisms seeded onto a coupon (λ).

The three most critical parameters to assessing survival in the model are shown in Figure 5: the *D*-value at 150 °C [δ(*T*_0_)], the *z*-value (*z*) and the mean number of microorganisms seeded onto a coupon (λ). The model identifies a *D*-value with mean 22.7 min and 95% credibility interval (17.5, 29.0), a *z*-value with mean 31.2°C (30.2, 32.4), and mean number of microorganisms seeded onto a coupon of 1.4 × 10^6^ (5.5 × 10^5^, 3.2 × 10^6^). We also observe strong correlations between the *D*-value and *z*-value (as expected), and no significant correlation between these parameters and the mean number of microorganisms seeded onto a coupon (also expected). Note that the *D*-value at 150°C is significantly lower than that estimated in Section 2.1 prior to performing high temperature HMR experiments. This could be an indicator of a lower *z*-value at lower temperatures and is an area for further research. The modeled *z*-values are in-line with expectations prior to performing experiments (which were between 30 and 50°C). Finally, the modeled predictions for the mean number of spores seeded onto a coupon are consistent with the control experiments performed.

**Figure 6:**
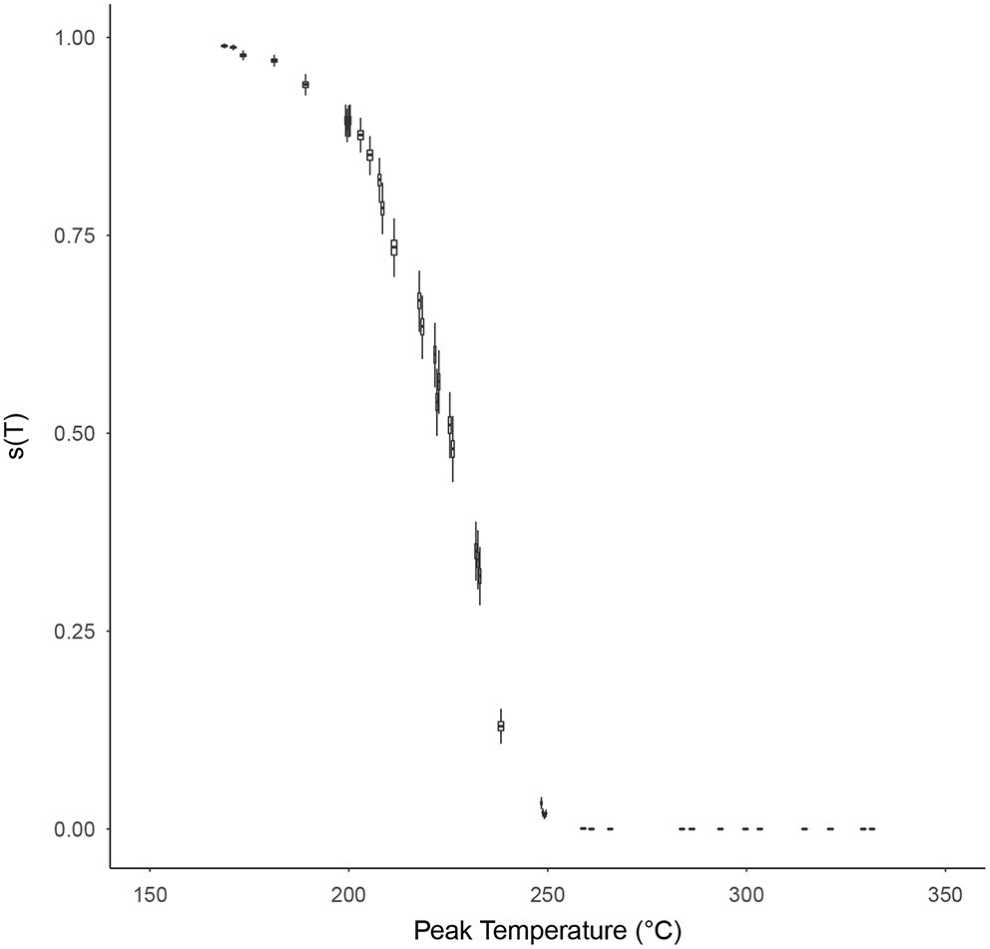
The probability of individual microorganism survival, *s*(*T*) (vertical axes), with respect to the peak temperature of the time-temperature profile, *T* (horizontal axes), for each survival ratio experiment, based on Equation (10). Box and whiskers show 50 and 95% credibility intervals, respectively, while horizontal lines accompanying each box-whisker show the mean value.

The probability of individual microorganism survival, *s*(*T*), with respect to the peak temperature of the time-temperature profile T was modeled for each high temperature HMR experiment, based on Equation (10), and is shown in Figure 6. The relationship between survival probability and peak temperature is non-linear with a significant drop in probability starting at around 200°C. After 250°C this probability becomes indiscernible from zero on a linear scale, and for peak temperatures >~290°C becomes vanishingly small (<10^−10^). Supplementary Figure 8 shows this survival plot in log-space for those wishing to discern the survival probabilities at these higher peak temperatures.

**Figure 7:**
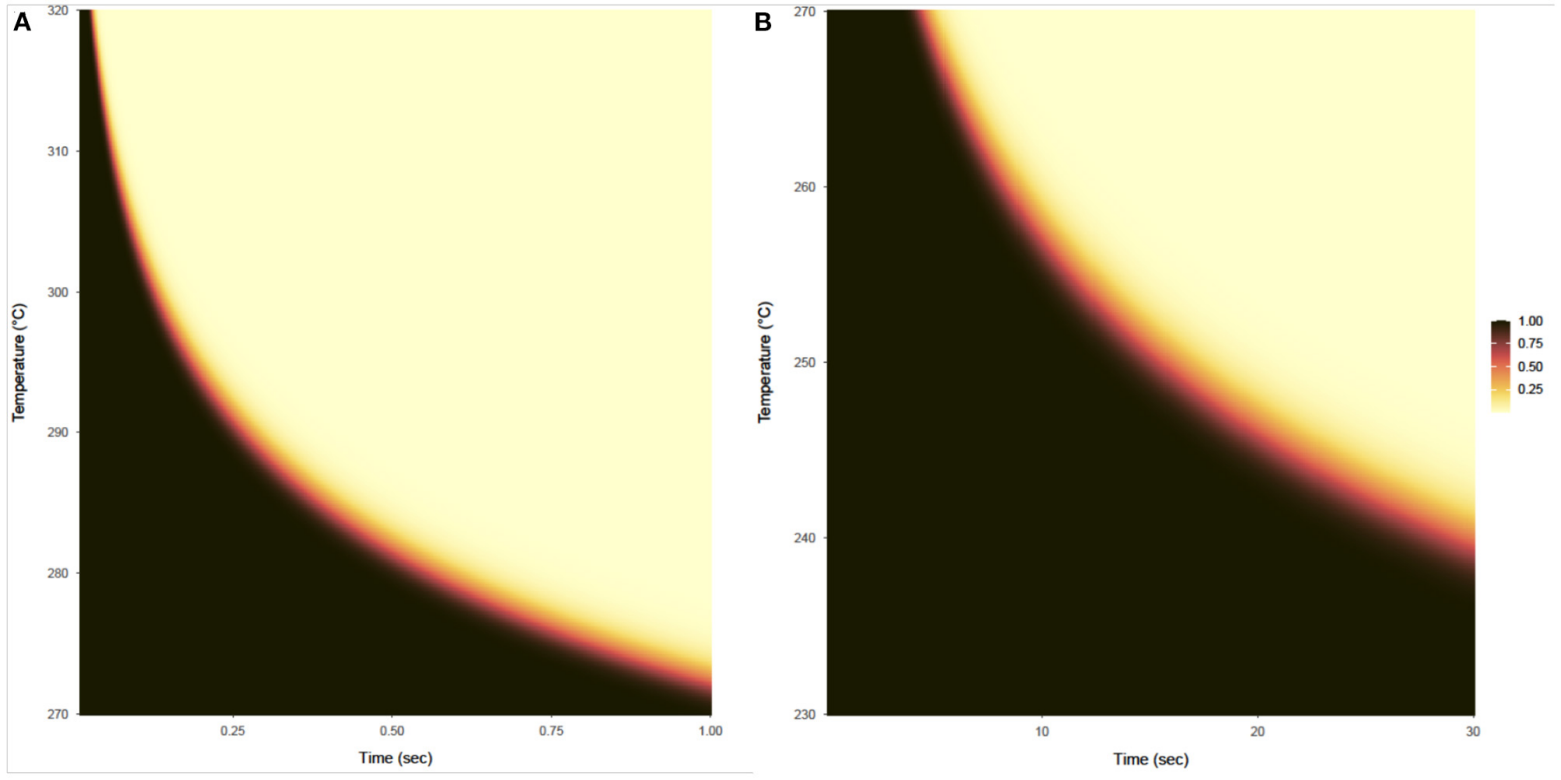
Probability that a product with a bioburden of 1 × 10^6^ is not sterile after being exposed to a given temperature (vertical axis) for a given time (horizontal axis). **(A)** Shows this probability for temperatures from 270 to 320°C and exposure times from 0.01 to 1 s, based on survival ratio and fraction-negative experiments and calculated using the expression on the left side of Equation (13). **(B)** Shows this probability for temperatures from 230 to 270°C for exposure times from 0.1 to 30 s, based on survival ratio experiments only and calculated using the expression on the left side of Equation (14).

**Figure 8:**
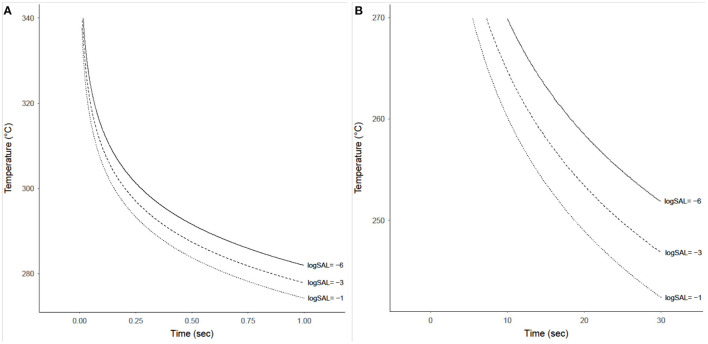
Time-temperature contours achieving sterility assurance levels (SAL) of 10^−1^, 10^−3^, and 10^−6^ for a product with an initial bioburden of 1 × 10^6^ prior to heat treatment. **(A)** Shows these contours for temperatures from 275 to 340°C and exposure times from 0.01 to 1 s. **(B)** Shows these contours for temperatures from 240 to 270°C and exposure times from 0.1 to 30 s, although these SAL contours only exist starting at ~7 s.

The probability that a product with a bioburden of 1 × 10^6^ is not sterile after being exposed to a given temperature is calculated using Equations (13) and (14), with results shown in Figures 7A,B for temperatures above 270°C and below 270°C, respectively. This probability is almost binary with respect to time and temperature, as can be seen by the rapid gradient change from black to yellow. The sterility assurance level (SAL) can be found within the gradient and used to generate a curve to determine time and temperature needed (Figure 8).

## 4. Discussion

The built-in overly-conservative specifications of the NASA provisions for sterility with HMR carries implications for future Planetary Protection implementation—for both flight missions and on-ground processing. The present study evaluated the survival of *B. canaveralius* 29669 heat-hardy spores at temperatures above 200°C and mathematically modeled the probability of spore survival to identify a refined Planetary Protection sterilization temperature specification. The *B. canaveralius* 29669 spores used in this study served to determine the NASA Procedural Requirements for heat sterilization values (originally NPR8020.12D and proceeded by NID 8020.109A) due to their ability to withstand high temperatures (Schubert and Beaudet, 2011; NASA Science Mission Directorate, 2017). By using the same spores, this experiment demonstrated that the 500°C for 0.5 s time and temperature for sterilization should be revised based on empirical data and modeling. For example, we predict for a bioburden of 1 × 10^6^ *B. canaveralius* 29669 spores, exposure to 292°C for 0.5 s achieves a SAL of 10^−6^.

The 6 kW high-heat infrared lamp set-up demonstrates the ability to test and record exposures at very high temperatures (>200°C) and millisecond intervals. The IR heating profile using the custom-machined titanium holder was obtained using a 0.914 emissivity found at known temperatures as well as with thermocouples spot-welded to the stainless steel coupons. The *B. canaveralius* 29669 spores were placed onto Kapton tape at a concentration predicted to produce a monolayer based on spore size and imaging (Figure 3), with limited overlap between the spores. Using this setup, a curve of organisms surviving heating versus the peak exposure temperature was produced as were videos with the infrared heating (and cooling) temperatures over the course of each exposure. In addition, a fraction-negative set of exposures along with their corresponding maximum temperatures and IR heating videos was also produced, with no organisms surviving temperatures above 268°C (Supplementary Table 1).

The processes and experimental results generated from the spore exposure experiments are captured, within a probabilistic framework, in the mathematical model developed to assess the probability of sterility. The comprehensive model considers how many spores are on the coupon prior to heat treatment and the survival and recovery efficiency of microorganisms from the coupon after heat treatment. The model was validated using the results from the survival ratio experiments and captured data dispersion well, with only 13% of experiments having actual observations falling outside the 95% credibility intervals of the model. Future analysis will test out-of-sample prediction strength.

The study conservatively demonstrates the likelihood of microorganism survival when exposed to high temperatures at short durations. The first element of conservatism is that this study exclusively used the heat tolerant *B. canaveralius* 29669 as the test organism. In a spacecraft assembly environment the microorganisms present are diverse, with many not being spore-formers and even fewer that have extreme heat tolerance. *B. canaveralius* 29669 was selected as the “worst-case” organism and parameters necessary to render its spores unable to germinate or replicate should apply to all non-heat-tolerant organisms as well. Next, the amount of spores tested on each coupon (~10^6^) is an over-estimate of the actual amount of spores present on any piece of spacecraft hardware. Smaller populations would need less time to attain the same SAL levels as larger populations. Given these conservative test parameters, the times and temperatures needed to sterilize sparsely distributed non-hardy microbial populations is likely to be even lower than the results presented in Figure 8. Therefore, while the results suggest a much less conservative time-temperature profile than the currently approved specification, it still represents an extreme scenario for spacecraft hardware conditions and sufficiently bounds the parameters needed for sterilization. Future work needs to be done to determine any margin that needs to be added in time and temperature to account for thermocouple sensitivity when implementing this for actual flight hardware, particularly at the higher temperatures (over 300°C) where there may be millisecond differences between sterility assurance levels of 10^-6^ and 10^-1^.

## 5. Conclusion

This study reports experimental evidence supporting a re-evaluation of the current Planetary Protection time and temperature overkill bioburden reduction requirement from 500°C for 0.5 s to a sterility curve where time to sterility can be calculated based on the given high-heat temperature (>200°C). The study demonstrates that results from the *B. canaveralius* 29669 survivor ratio and fraction negative experiments can be used to mathematically model SAL curves.

HMR at high temperatures is often incompatible with spacecraft hardware and poses a limitation to the application of it as a bioburden control method. While in-flight, certain spacecraft zones may also be subject to rapid exposures to high temperature from propellant ignition (e.g., during powered descent), that is currently not accounted for when determining lethality of bioburden. Reassessment of the sterility specifications to a “sterility curve” rather than a point requirement (500°C for 0.5 s) allows for a wider range of time and temperatures that could be compatible with spacecraft materials and support more pre-flight HMR. This result will increase microbial reduction credit that can be taken at lower heat microbial reduction temperatures in Planetary Protection relevant interplanetary missions.

First, seven isolates of *B. canaveralius* 29669 spores, the same extremophile strain used to obtain the NPR 8020.12D (now NID 8020.109A) Planetary Protection heating parameters, were exposed to 150°C heat for 30 min. Of these cultures, the *B. canaveralius* 29669 strain most closely matching the *D*-value (39.7 min at 150°C) from the NPR 8020.12D culture used to set the heating values was used. A 5080 6 kW halogen infrared lamp was then employed and characterized for its ability to precisely increase the temperature of half inch long stainless steel coupons to 350°C in under 10 s. A T650sc infrared camera was also incorporated to accurately and quickly heat spores. The emissivity of the *B. canaveralius* 29669 spores on Kapton-taped stainless steel coupons was determined to be 0.914 using a known temperature, calibrating the T650sc infrared camera. Approximately 4 × 10^6^ *B. canaveralius* 29669 spores were then heated to peak temperatures of 184.7 to 334.2°C on the scale of seconds, using the 5080 halogen lamp, and no organisms survived temperatures above 268°C. Fraction negative (binary, growth/no-growth) tests were performed to confirm the ability of the heat to sterilize the *B. canaveralius* 29669 spores. Finally, the ramp rate for the heater did not correlate to spore death from 35.72 to 50.71°C/s.

## Data Availability Statement

The raw data supporting the conclusions of this article will be made available by the authors, without undue reservation.

## Author Contributions

ZD and MD contributed equally to carrying out the research. ZD performed the heat microbial reduction experiments, the analysis, designed the figures, and drafted the manuscript sections regarding experimental data acquisition, while MD contributed the mathematical modeling analysis, figures, and manuscript sections using Stan software (Stan Development Team, 2021) in RStudio (RStudio Team, 2021). EK contributed to the initial spore characterization research and the initial experiment design. SR supplied the IR camera as well as his IR image acquisition knowledge and assisted in the emissivity measurements. BC wrote and critically reviewed the manuscript. LG conducted experimental recovery experiments, provided inputs to the modeling, and wrote and critically reviewed the manuscript. All authors contributed to the research design at some point during the project.

## Funding

The research was carried out at the Jet Propulsion Laboratory, California Institute of Technology, under a contract with the National Aeronautics and Space Administration (80NM0018D0004).

## Author Disclaimer

This manuscript was prepared as an account of work sponsored by NASA, an agency of the US Government. The US Government, NASA, California Institute of Technology, Jet Propulsion Laboratory, and their employees make no warranty, expressed or implied, or assume any liability or responsibility for the accuracy, completeness, or usefulness of information, apparatus, product, or process disclosed in this manuscript, or represents that its use would not infringe upon privately held rights. The use of, and references to any commercial product, process, or service does not necessarily constitute or imply endorsement, recommendation, or favoring by the US Government, NASA, California Institute of Technology, or Jet Propulsion Laboratory. Views and opinions presented herein by the authors of this manuscript do not necessarily reflect those of the US Government, NASA, California Institute of Technology, or Jet Propulsion Laboratory, and shall not be used for advertisements or product endorsements. Reference herein to any specific commercial product, process, or service by trade name, trademark, manufacturer, or otherwise, does not constitute or imply its endorsement by the United States Government or the Jet Propulsion Laboratory, California Institute of Technology. Government sponsorship acknowledged.

## Conflict of Interest

The authors declare that the research was conducted in the absence of any commercial or financial relationships that could be construed as a potential conflict of interest.

## Publisher's Note

All claims expressed in this article are solely those of the authors and do not necessarily represent those of their affiliated organizations, or those of the publisher, the editors and the reviewers. Any product that may be evaluated in this article, or claim that may be made by its manufacturer, is not guaranteed or endorsed by the publisher.

## Footnotes

^1^*A word on notation used in this document*: The notation *p*(*x*∣*y*) will be used to denote the conditional probability function of a random variable (or random vector) *X* taking the value *x* given another random variable (vector) *Y* takes the value *y*. When *X* is discrete, *p*(*x*∣*y*) will be understood to be a probability mass function; when *X* is continuous, *p*(*x*∣*y*) will be understood to be a probability density function. When we are interested in evaluating this probability function at a specific value *x*_0_, then we will write *p*(*x* = *x*_0_∣*y*). Finally, the unconditional probability function of a random variable (vector) *X* will be denoted as *p*(*x*).
