# Supplementary material for: Establishing Sterility Assurance for Bacillus canaveralius 29669 Spores Under High Heat Exposure
Source: Front Microbiol. 2022 Jul 11;13:909997. doi: 10.3389/fmicb.2022.909997 (PMC9309537; doi:10.3389/fmicb.2022.909997)
Supplement: Supplementary file 1 [file Data_Sheet_1.PDF]

frontiers

Supplementary Material

1 SUPPLEMENTARY METHODS

1.1 The Survival Function

Microorganism survival has been shown to decay exponentially with increased time at given temperatures. This is typically due to high enough temperatures leading to failure of cell membranes and denaturing of proteins, breaking the chemical bonds that allow them to hold their shape and perform cell-critical functions. Suppose the function relating the D-value, δ, of a given species, to temperature, T, is continuous for all $T \in (T_0, T_1)$ such that $T_0 < T_1$, and differentiable at all but potentially a finite number of points on this interval. It is observed in the microbial reduction community that a constant change in temperature tends to be related to orders-of-magnitude changes in the D-value for a given microorganism. More generally, suppose this relationship can be described by the Riemann integrable function, $g(T)$. When $\delta(T)$ is differentiable at T, this relationship can be expressed by the differential equation

$$\frac{\mathrm{d}\left[\ln\left(\delta(T)\right)\right]}{\mathrm{d}T} = -g(T)\,, \tag{S1}$$

where the negative sign, by convention, is utilized on the right side of the equation. Integrating both sides of the equation between T_0 and T_1, and applying the Fundamental Theorem of Calculus,[1] we have

$$\ln\left(\delta(T_1)\right) - \ln\left(\delta(T_0)\right) = -\int_{T_0}^{T_1} g(T)\,\mathrm{d}T\,, \tag{S2}$$

and applying rules of logarithms and exponents gives us

$$\frac{\delta(T_1)}{\delta(T_0)} = e^{-\int_{T_0}^{T_1} g(T)\,\mathrm{d}T}\,. \tag{S3}$$

Now, letting $N(T,t)$ be the number of surviving microorganisms after time t when exposed to temperature T, the partial differential equation

$$\frac{\partial\left[\ln(N(T,t))\right]}{\partial t} = -b(T) \tag{S4}$$

expresses how the survival ratio is expected to change with respect to time. In practice, $b(T)$ is a non-zero constant for each T, and the value $\ln(10)/b(T)$ is the D-value, $\delta(T)$, given by Equation (S3). Note that b does not depend on the time t.

[1] Note that a finite number of points at which $\delta(T)$ is not differentiable can be handled by applying Equation (S1) to each interval where $\delta(T)$ is differentiable, integrating over each of these intervals individually, as in Equation (S2), and summing these integrals. Essentially, these finite points of non-differentiability have measure zero, and so contribute nothing to the integral over the entire interval.

Similar to how we solved the differential equation in (S1), we solve the differential equation in (S4) when $t \in (t_0, t_1)$ for a fixed temperature, T^*, to get the expected survival ratio

$$\frac{N(T^*, t_1)}{N(T^*, t_0)} = e^{-\Delta t\, b(T^*)} \tag{S5}$$

where $\Delta t = t_1 - t_0$, and combining this with Equation (S3), gives us

$$\begin{aligned} \frac{N(T^*, t_1)}{N(T^*, t_0)} &= \exp\left\{ -\Delta t \frac{\ln(10)}{\delta(T^*)} \right\} \\ &= \exp\left\{ -\Delta t \frac{\ln(10)}{\delta(T_0)} e^{\int_{T_0}^{T^*} g(T)\, \mathrm{d}T} \right\} \end{aligned} \tag{S6}$$

where T_0 is a fixed reference temperature chosen from the interval over which the z-value relationship from Equation (S1) holds (typically taken to be the infimum of values on this interval). Two important observations should be made at this point:

1. Since the D-value does not depend on the exposure time to temperatures prior to time t_0, the expected survival ratio depends only on the time duration, Δt, exposed to temperature T^*. Put another way, this model implies that the survival ratio does not depend on how much time a set of microorganisms may have previously survived a heat treatment. When evaluating whether these microorganisms will survive the next time interval of heat treatment, all that matters is the temperature experienced on, and the duration of, that next time interval.
2. Because we can assume that individual microorganism survival events are independent of one another, and that all microorganisms share the same individual probability of survival, the expected survival ratio from Equation (S6) can be interpreted as a probability. To see this, suppose there are $N(T^*, t_0) = n$ microorganisms that have survived up to time t_0 of the heat treatment and let $s(T^*, \Delta t)$ be the probability that an individual microorganism survives exposure to a temperature T^* for time duration Δt after time t_0. Then it follows from our assumptions that the number of survivors follows a binomial distribution with expectation $N(T^*, t_0 + \Delta t) = n \times s(T^*, \Delta t)$. Solving for $s(T^*, \Delta t)$, we have $s(T^*, \Delta t) = \frac{N(T^*, t_0 + \Delta t)}{n}$, which is the survival ratio when the number of microorganisms at time t_0 of heat treatment is known.

Hence, from the discussion in (2) above, the probability that an individual microorganism survives when exposed to temperature, T^*, for time duration, Δt, is

$$s(T^*, \Delta t) = \exp\left(-\Delta t \frac{\ln(10)}{\delta(T_0)} e^{\int_{T_0}^{T^*} g(T)\, dT} \right), \tag{S7}$$

and from the discussion in (1) above, this probability is well defined because it is memoryless; i.e. the survival of an individual microorganism over any time interval on the time-temperature profile depends on the length of the time interval, but not on how much time the microorganism has already survived up to that time.

In order to connect the survival probability with a specified time-at-temperature during heating events of interest, let $T : [t_0, \infty) \to \mathbb{R}$ be a real-valued function referred to as the time-temperature profile, where $T(t)$ is the temperature at time t for a given set of hardware. We will also assume this function is differentiable at every time $t \in [t_0, \infty)$, and, given applications of this work, we will restrict our attention

to finite sub-intervals $[t_0, t_\mathrm{f})$ within $[t_0, \infty)$ where temperatures exceed a reference temperature T_0 for which microorganism mortality dominates any kind of growth or replication.

Now, partition the time interval $[t_0, t_\mathrm{f})$ into n intervals of size $\Delta t > 0$ and let $T_i^* = \sup\{T(t) \mid t \in (t_{i-1}, t_i)\}$. We will denote this partition by $\mathfrak{P}_n$. As n goes to ∞ and Δt goes to 0, this partition will be refined further and further, and we will say that $\mathfrak{P}_n$ goes to $\mathfrak{P}_\infty$ when we take limits.

The probability that a microorganism survives exposure to the time-temperature profile, $T(t)$, on the interval, $[t_0, t_\mathrm{f})$, is approximately the probability that a microorganism survives exposure to the temperature T_i^* on each time interval of length Δt:

$$\begin{aligned} s(T(t))\Big|_{t_0}^{t_\mathrm{f}} &\approx \prod_{i=1}^{n} s(T_i^*, \Delta t) \\ &= \prod_{i=1}^{n} \exp\left(-\Delta t \frac{\ln(10)}{\delta(T_0)} e^{\int_{T_0}^{T_i^*} g(T)\,\mathrm{d}T}\right) \\ &= \exp\left(-\frac{\ln(10)}{\delta(T_0)} \sum_{i=1}^{n} e^{\int_{T_0}^{T_i^*} g(T)\,\mathrm{d}T} \Delta t\right) . \end{aligned} \tag{S8}$$

Taking limits as the partition is refined gives the solution

$$\begin{aligned} s(T(t))\Big|_{t_0}^{t_\mathrm{f}} &= \lim_{\mathfrak{P}_n \to \mathfrak{P}_\infty} \exp\left(-\frac{\ln(10)}{\delta(T_0)} \sum_{i=1}^{n} e^{\int_{T_0}^{T_i^*} g(T)\,\mathrm{d}T} \Delta t\right) \\ &= \exp\left(-\frac{\ln(10)}{\delta(T_0)} \lim_{\mathfrak{P}_n \to \mathfrak{P}_\infty} \sum_{i=1}^{n} e^{\int_{T_0}^{T_i^*} g(T)\,\mathrm{d}T} \Delta t\right) \\ &= \exp\left(-\frac{\ln(10)}{\delta(T_0)} \int_{t_0}^{t_\mathrm{f}} e^{\int_{T_0}^{T(t)} g(T)\,\mathrm{d}T}\,\mathrm{d}t\right) , \end{aligned} \tag{S9}$$

where the first equality follows by continuity of our probability measure; the second equality follows from the continuity of the exponential function; and the third equality follows since g is Riemann integrable and $T(t)$ is differentiable.

Note that this formulation includes the case of a constant z-value assumed in this study. In this case, we have, for all temperatures $T \geq T_0$ for which microorganism mortality is assumed,

$$\frac{\mathrm{d}\left[\ln\left(\delta(T)\right)\right]}{\mathrm{d}T} = -g(T) = -c\,, \tag{S10}$$

where c is a positive real number. Hence, the z-value, z, is equal to $\ln(10)/c$, and

$$\begin{aligned} s(T) \equiv s(T(t))\Big|_{t_0}^{t_\mathrm{f}} &= \exp\left(-\frac{\ln(10)}{\delta(T_0)} \int_{t_0}^{t_\mathrm{f}} e^{\int_{T_0}^{T(t)} c\,\mathrm{d}T}\,\mathrm{d}t\right) \\ &= \exp\left(-\frac{\ln(10)}{\delta(T_0)} \int_{t_0}^{t_\mathrm{f}} e^{\ln(10)[T(t)-T_0]/z}\,\mathrm{d}t\right) . \end{aligned} \tag{S11}$$

This constant z-value model is the simplest and most used in practice. However, more complex models, such as when the z-value varies linearly or quadratically with T, may also be reasonable. Investigating different ways in which the z-value may depend on the temperature T and insights provided by thermodynamics and chemical kinetics (e.g. Arrhenius and Eyring equations) are currently being investigated but are beyond the scope of this study. Finally, Figure 7 in the main article shows the behavior of this Equation (S11) on a linear scale. Here we also include Figure S8 which is the same data but plots the common logarithm of $s(T)$ with respect to the peak temperature of the time-temperature profile, T. This makes visible the boxplots at higher temperatures (>250 °C) and the drastic decrease in the probability of individual microorganism survival predicted by the model when exposed to peak temperatures above ∼290 °C in the experiments conducted as part of this study.

1.2 Prior Distributions for Parameters

Studies performed prior to performing HMR experiments allowed initial marginal distributions to be developed for each parameter of the model. Controls performed for this study to assess the inoculation level of coupons provided a distribution of the mean number of microorganisms seeded onto a coupon, λ. Another set of controls to assess extraction efficiency from the coupons provided an initial distribution for ϕ. Experiments performed for spore strain selection discussed in Section 2.1 with results shown Figure S5 provided a distribution for the D-value. Current handbooks in the sterilization community (European Space Agency for the members of European Cooperation for Space Standardization, 2013; Moldenhauer, 2019) provide a range of estimates for dry heat z-values. Finally, initial distributions for θ_j are based on the dilution magnitude and pour fraction. The intent of these distributions is to reasonably bound the state of knowledge of these parameter values. Observations from the HMR experiments performed in this study are of sufficient number such that minor to moderate differences as to how this prior state of knowledge is quantified has little effect on the model results.

1.2.1 Prior Distribution for λ, the Mean Number of ATCC-29669 Spores Seeded on a Coupon

The prior distribution for the mean number of spores seeded onto a coupon used in this experiment is

$$\lambda \sim \text{Lognormal}(\mu_\lambda, \sigma_\lambda)\,, \tag{S12}$$

where $\mu_\lambda = 14.85$ and $\sigma_\lambda = 0.48$. This implies a mean number of 3.2×10^6 spores per coupon, with 99% of the probability falling between 8.2×10^5 and 9.9×10^6 spores per coupon. Parameter values for this distribution were estimated from control experiments consisting of five replicates, with three dilutions (10^{-7}, 10^{-8} and 10^{-9}) per replicate.

1.2.2 Prior Distribution for $\delta(T_0)$, the D-value for *Bacillus* ATCC-29669 Spores at 150 °C

The prior distribution for the D-value of *Bacillus* ATCC-29669 spores at 150 °C is

$$\delta(T_0) \sim \text{Lognormal}(\mu_{\delta(T_0)}, \sigma_{\delta(T_0)})\,, \tag{S13}$$

where $\mu_{\delta(T_0)} = 7.65$ and $\sigma_{\delta(T_0)} = 0.1$. This implies a mean D-value of ∼2100 sec (35 min), with 99% of the probability falling between ∼1600 sec (27 min) and ∼2700 sec (45 min). Parameter values for this distribution were estimated from the data presented in Figure S5.

1.2.3 Prior Distribution for z, the z-value for *Bacillus* ATCC-29669 Spores

The prior distribution for the z-value of *Bacillus* ATCC-29669 spores is

$$z \sim \text{Lognormal}(\mu_z, \sigma_z)\,, \tag{S14}$$

where $\mu_z = 3.66$ and $\sigma_z = 0.25$. This implies a mean z-value of 40 °C, with 99% of the probability falling between 20 °C and 74 °C. This is consistent with current handbooks in the sterilization community (European Space Agency for the members of European Cooperation for Space Standardization, 2013; Moldenhauer, 2019) while allowing for a broad range of uncertainty reflecting the lack of data to inform this parameter.

1.2.4 Prior Distribution for ϕ, the probability that an individual *Bacillus* ATCC-29669 spore that survived heat treatment is extracted from the coupon and survives extraction

The probability that an individual *Bacillus* ATCC-29669 spore that survived heat treatment is extracted from the coupon into water medium and survives extraction is

$$\phi \sim \text{Beta}(\alpha_\phi, \beta_\phi)\,, \tag{S15}$$

where $\alpha_\phi = 8.75$ and $\beta_\phi = 16.25$. This is equivalent to a mean extraction probability for an individual microorganism of 0.35, with a dispersion equal to 25. The dispersion was set in a way such that 99% of the probability lies roughly ± 0.25 around the mean. Parameter values for this distribution were estimated from control data assessing extraction efficiency from the Kapton covered steel coupons used in this study.

1.2.5 Prior Distribution for θ, the probability that an individual viable *Bacillus* ATCC-29669 spore is transferred from the original solution in which it was extracted to a dilution, plated and produces a CFU

The prior distribution associated with the probability that an individual viable *Bacillus* ATCC-29669 spore is transferred from the original extraction solution to a dilution of magnitude i, to a petri dish to then produce a CFU is

$$\theta_i \sim \text{Beta}(1, 10^i - 1)\,, \tag{S16}$$

where $i = 1, \ldots, 5$. This results in the mean of θ_i being equal to 10^{-i}, with 99% of the probability falling roughly between $5 \times 10^{-i-3}$ and 5×10^{-i}.

2 SUPPLEMENTARY TABLES AND FIGURES

2.1 Figures

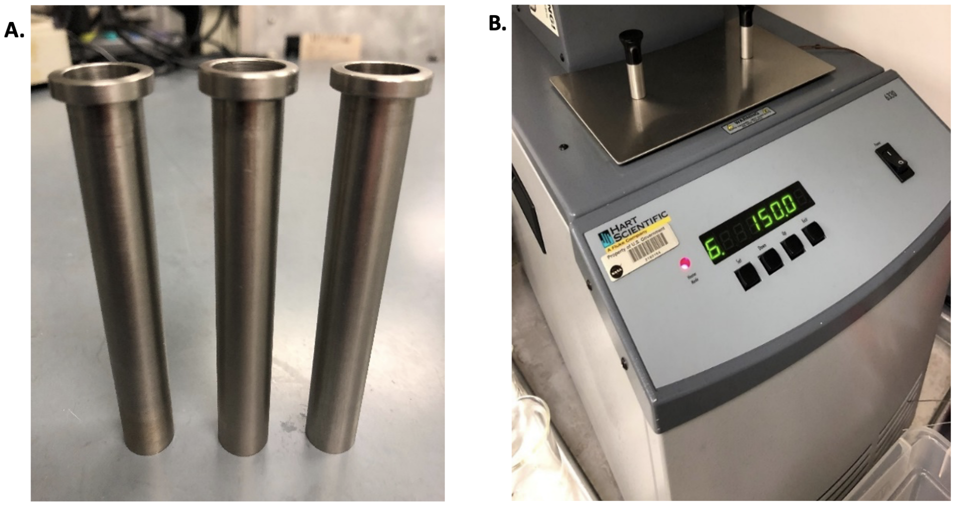

Figure S1. Images of the equipment used for exposing spores at 150 °C for dry heat resistance characterization. **(A)** Thermal spore exposure vessels (TSEVs) and **(B)** the Hart Scientific silicon oil calibration bath.

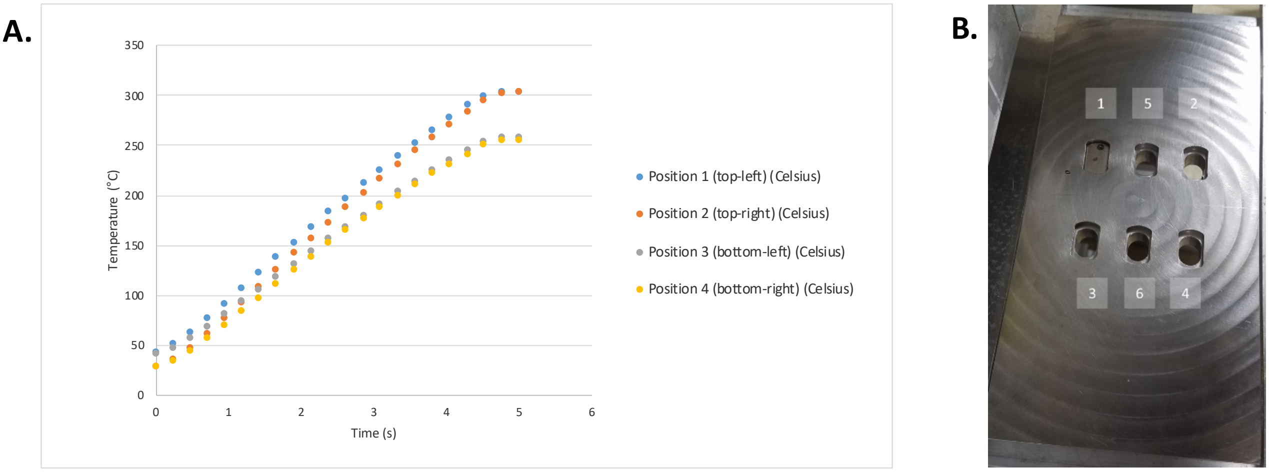

Figure S2. Infrared heat lamp characterization. Graphic of **(A)** heating ramp differences depending on **(B)** the position of the stainless steel coupon in the titanium holder. Thermocouples spot-welded to blank coupons were used to acquire the temperatures.

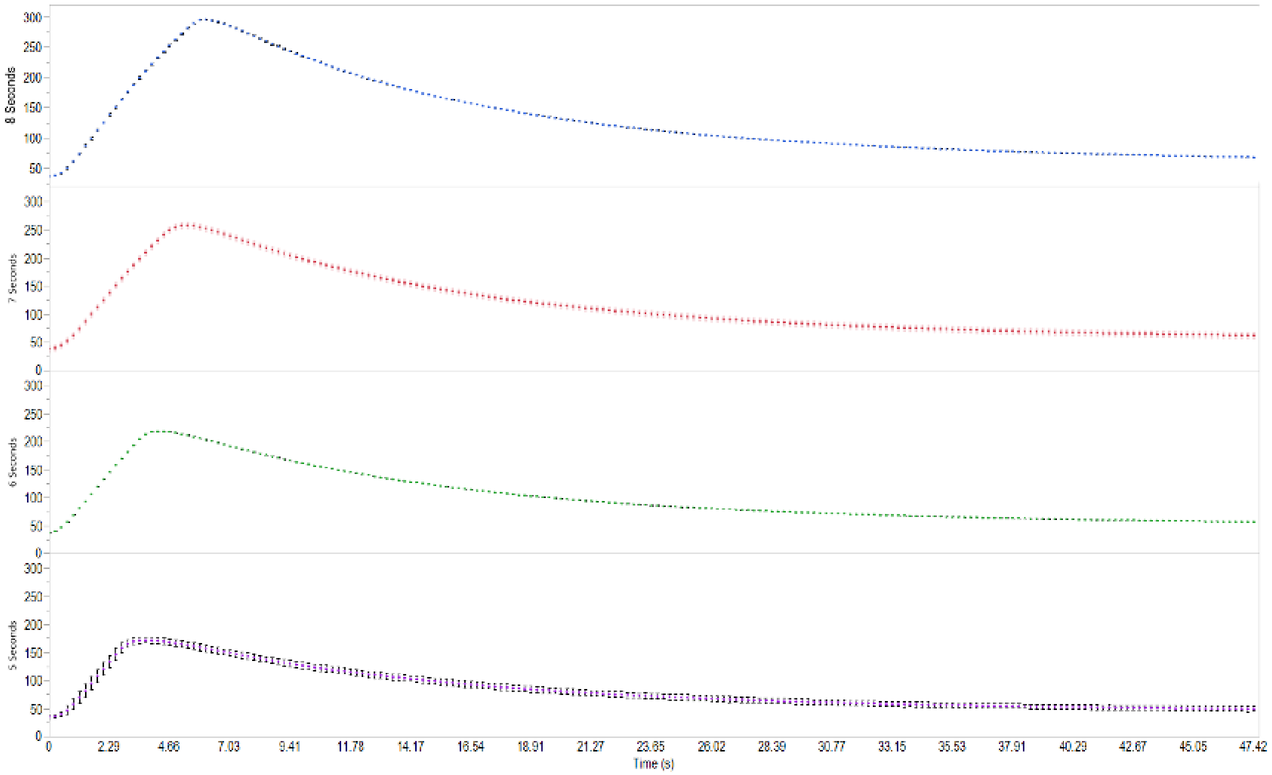

Figure S3. IR heater temperature-over-time data measured in Position 1 of the custom titanium holder with a K-type thermocouple. Heating times shown were measured from the time the IR lamp was turned on. There is a slight delay before heating begins after the lamps are turned on, which can be seen in the graph.

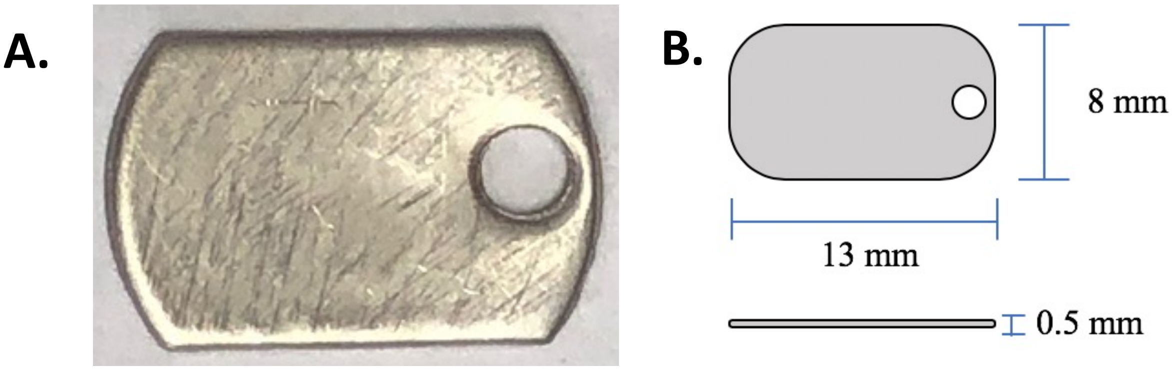

Figure S4. Spore coupon description. **(A)** Image of stainless steel coupon. **(B)** Graphic of coupon with corresponding dimensions.

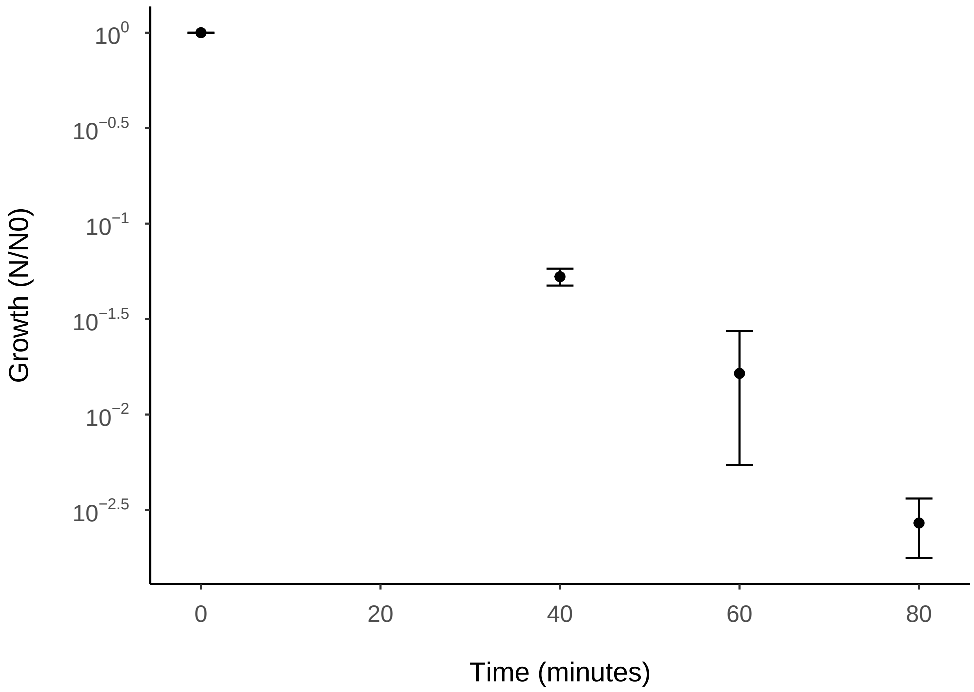

Figure S5. Survival plot of spore batch M4-6 at 150°C and a starting population of 1.5x10^6 spores per microliter. The spores were placed under vacuum (controlled humidity) conditions, and were evacuated to below 1.5 Torr.

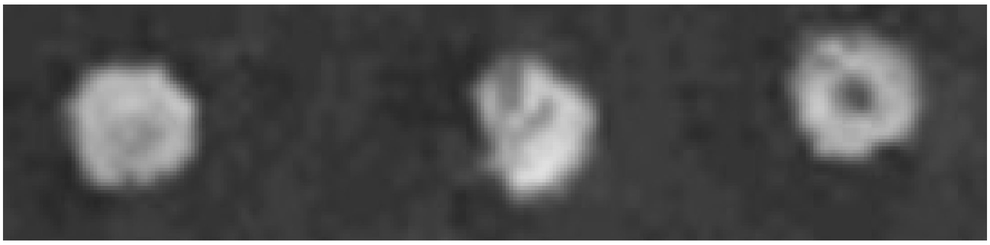

Figure S6. A zoomed-in image of spores spotted on a Kapton taped stainless steel coupon. The spores were dried overnight before plating. The left and middle spots exhibit no visible coffee ring effect, but the right spot does show a coffee ring effect. Measuring the diameter of the right spot as well as the middle spore-vacant region, then calculating the area of each circle, showed that the coffee ring effect only resulted in a 10% loss of surface area. As the coffee ring effect is not seen in the other spots the coffee ring effect on spore spots is assumed 10% or less.

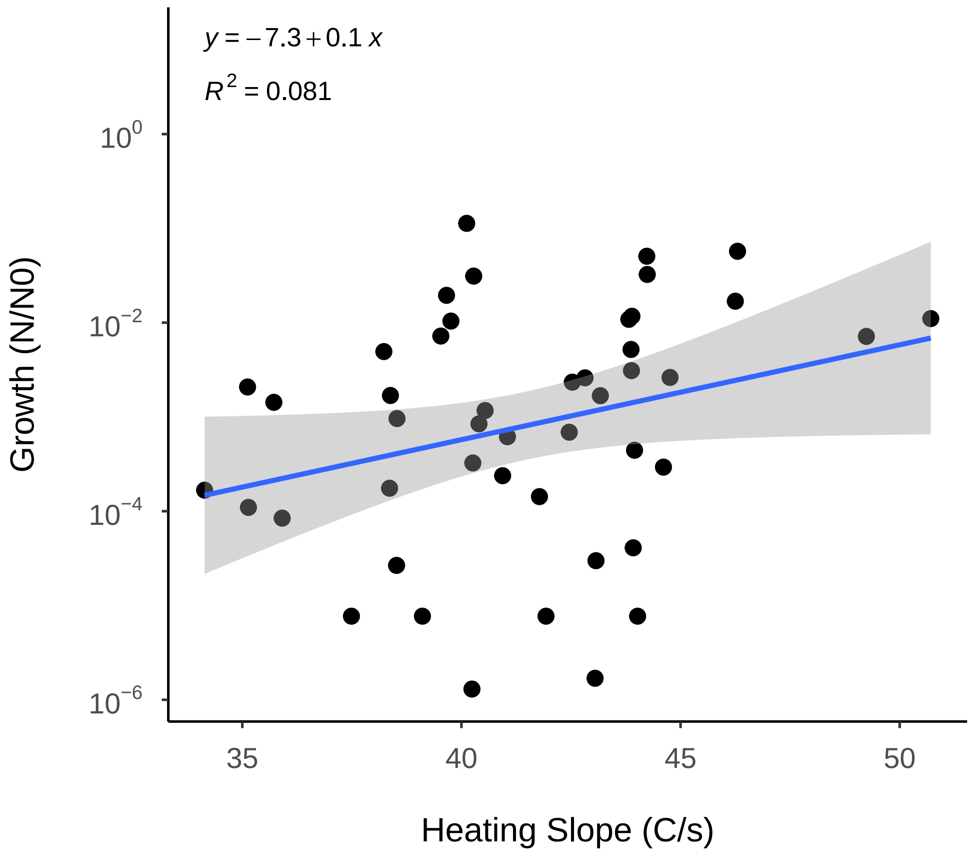

Figure S7. Characterization of the coupon heating slope (ramp up) and its effect on ATCC-29669 cell death. Growth is measured as CFU observed following heat exposure divided by the starting population seeded onto the coupons (y-axis). he ramp from room temperature to the maximum temperature was assumed linear to acquire the heating slope (x-axis). The blue line represents best fit in a log-linear model while the shaded area shows the 95% confidence interval. There is no correlation between coupon heating slope and spore survival (R^2 = 0.081, p-value = 0.06).

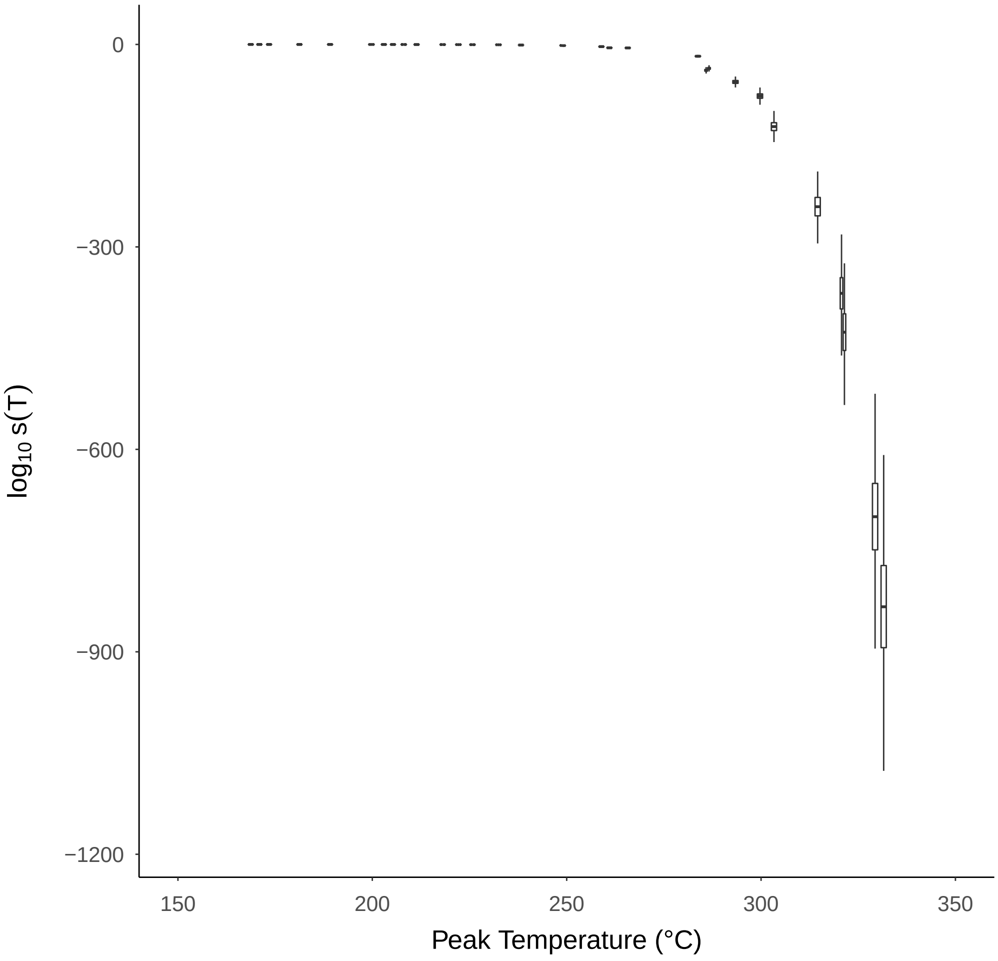

Figure S8. The common logarithm (base 10) of the probability of individual microorganism survival (vertical axes), with respect to the peak temperature of the time-temperature profile (horizontal axes), for each HMR experiment, based on Equation (S11). Box and whiskers show 50% and 95% credibility intervals, respectively, while horizontal lines accompanying each box-whisker show the mean value.

2.2 Tables

Table S1. HMR experimental results.

Run	Replicate	Dilution Magnitude	CFU	PeakTemp (degC)	Experiment Type
1	1	2	555	207.8	Survival Ratio
2	1	3	11	219.8	Survival Ratio
3	1	4	24	174.5	Survival Ratio
4	1	2	25	228	Survival Ratio
5	1	4	13	184.7	Survival Ratio
7	1	3	38	203.4	Survival Ratio
8	1	4	44	189.7	Survival Ratio
9	1	1	135	226.6	Survival Ratio
12	1	4	4	218	Survival Ratio
13	1	4	87	185.4	Survival Ratio
15	1	2	53	237.4	Survival Ratio
16	1	4	13	254.2	Survival Ratio
17	1	4	15	205.4	Survival Ratio
18	1	4	39	197	Survival Ratio
19	1	4	9	225.2	Survival Ratio
20	1	2	11	253.8	Survival Ratio
21	1	4	25	201.1	Survival Ratio
22	1	4	0	256.4	Survival Ratio
23	1	4	2	229.3	Survival Ratio
24	1	4	8	175.4	Survival Ratio
37	1	2	22	233.3	Survival Ratio
37	2	3	1	233.3	Survival Ratio
37	3	1	230	233.3	Survival Ratio
38	1	1	13	250	Survival Ratio
38	2	2	5	250	Survival Ratio
39	1	4	0	286.4	Survival Ratio
40	1	4	8	206.1	Survival Ratio
40	2	3	87	206.1	Survival Ratio
41	1	2	68	223.3	Survival Ratio
41	2	3	2	223.3	Survival Ratio
41	3	4	3	223.3	Survival Ratio
42	1	3	7	221.9	Survival Ratio
42	2	2	60	221.9	Survival Ratio
43	1	4	7	207.8	Survival Ratio
43	2	3	100	207.8	Survival Ratio
44	1	3	7	225.5	Survival Ratio
44	2	2	97	225.5	Survival Ratio
45	1	5	1	213.2	Survival Ratio
45	2	4	4	213.2	Survival Ratio
45	3	3	25	213.2	Survival Ratio
46	1	3	3	233	Survival Ratio
46	2	2	20	233	Survival Ratio
46	3	1	178	233	Survival Ratio
47	1	3	7	240.6	Survival Ratio
47	2	2	16	240.6	Survival Ratio
47	3	1	164	240.6	Survival Ratio
48	1	1	1	264.6	Survival Ratio
49	1	NA	0	268	Fraction Negative
50	1	NA	0	326.4	Fraction Negative
51	1	NA	0	334.2	Fraction Negative
52	1	NA	0	307.2	Fraction Negative
53	1	NA	0	296.3	Fraction Negative
54	1	NA	0	315	Fraction Negative
55	1	NA	0	311.6	Fraction Negative
56	1	NA	0	294.6	Fraction Negative
57	1	NA	0	322.9	Fraction Negative
58	1	NA	0	332.2	Fraction Negative
59	1	NA	0	294	Fraction Negative

REFERENCES

European Space Agency for the members of European Cooperation for Space Standardization (2013). Space Product Assurance; Dry Heat Bioburden re-duction for flight hardware.

Moldenhauer, J. (2019). *Disinfection and Decontamination: A Practical Handbook* (CRC Press)
